# Histidine triad nucleotide-binding protein 2 attenuates metabolic dysfunction-associated steatotic liver disease through NAD^+^-dependent sirtuin-3 activation

**DOI:** 10.1038/s12276-025-01445-w

**Published:** 2025-05-01

**Authors:** Qinqiu Wang, Yanjun Guo, Shenghui Chen, Zhening Liu, Xinyu Wang, Hangkai Huang, Qi-en Shen, Ling Yang, Meng Li, Youming Li, Chaohui Yu, Chengfu Xu

**Affiliations:** Department of Gastroenterology, the First Affiliated Hospital, Zhejiang University School of Medicine, Hangzhou, China

**Keywords:** Cell biology, Metabolic disorders

## Abstract

Metabolic dysfunction-associated steatotic liver disease (MASLD) is the most common chronic liver disease, but its pathogenesis is unclear. Here we focus on histidine triad nucleotide-binding protein 2 (HINT2), which is expressed in the mitochondria and is involved in hepatic lipid metabolism and mitochondrial protein acetylation. The expression of HINT2 is downregulated in MASLD. HINT2 inhibits free fatty acid-induced lipid accumulation and impairs mitochondrial function in hepatocytes. *Hint2* knockout exacerbates diet-induced hepatic steatosis, inflammation, fibrosis and mitochondrial damage in mice. The overexpression of *Hint2* attenuates these alterations. Mechanistically, HINT2 regulates mitochondrial protein acetylation via SIRT3; HINT2 enhances the NAD^+^-dependent activation of sirtuin-3 (SIRT3) by promoting the mitochondrial influx of NAD^+^ through solute carrier family 25 member 51 (SLC25A51), thus ameliorating MASLD. Moreover, the downregulation of HINT2 in MASLD is due to YTH N^6^-methyladenosine RNA binding protein 1 (YTHDF1)-mediated regulation. Our results suggest that HINT2 may be an important therapeutic target for MASLD.

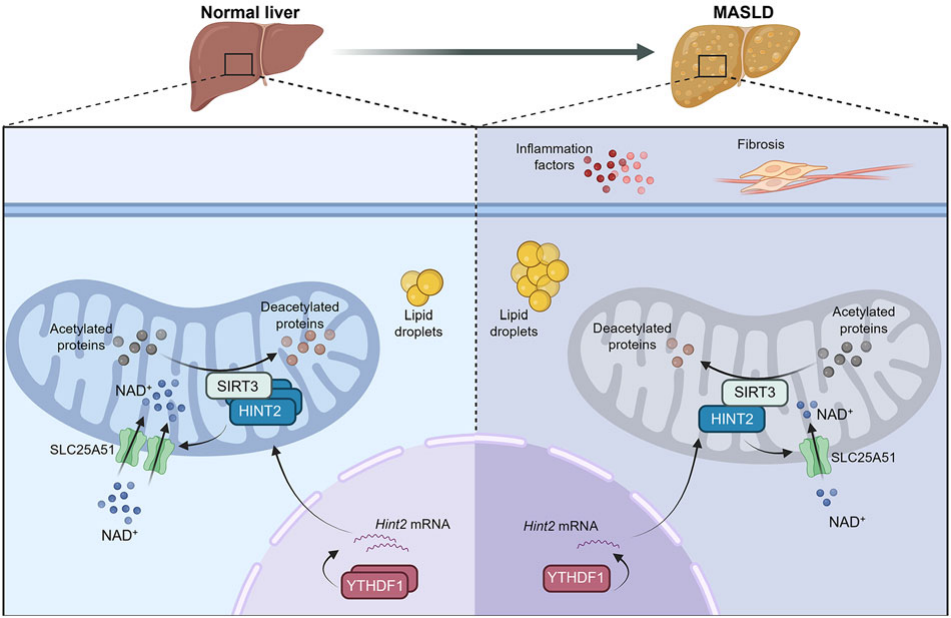

## HINT2 protein regulates liver fat metabolism and health

Metabolic dysfunction-associated steatotic liver disease (MASLD) is a common liver condition linked to other health issues such as heart disease and diabetes. Researchers found that a protein called HINT2, which is less active in patients with MASLD, might be important. They studied mice and cells to see how HINT2 affects liver health. The researchers used mice with and without HINT2 and fed them different diets to mimic MASLD. They found that mice lacking HINT2 had worse liver problems, while those with more HINT2 had healthier livers. HINT2 seems to help another protein, SIRT3, work better by increasing levels of NAD^+^ in the liver. The study shows that boosting HINT2 could protect against liver damage in MASLD. This research suggests that targeting HINT2 might be a new way to treat MASLD in the future.

This summary was initially drafted using artificial intelligence, then revised and fact-checked by the author.

## Introduction

Metabolic dysfunction-associated steatotic liver disease (MASLD) is currently the most common chronic liver disease worldwide, and its spectrum includes simple fatty liver disease, steatohepatitis, cirrhosis and end-stage liver disease^1^. MASLD is often associated with comorbidities, including cardiovascular disease, type 2 diabetes and stroke, and is emerging as a public health problem^2–4^. Therapies targeting the pathogenesis of MASLD are yet to be identified^5,6^. Therefore, there is an urgent need to identify specific targets for MASLD.

Impairments in mitochondrial structure and function are important triggers of MASLD^7^. *Hint2*-deficient mice reportedly exhibit respiratory dysfunction in hepatocytes, hepatic lipid accumulation and aberrant glucose metabolism^8^. *Hint2* deficiency also enhances the acetylation of hepatic mitochondrial proteins and decreases NAD^+^-dependent glutamate dehydrogenase and short-chain 3-hydroxyacyl-CoA dehydrogenase activities^8^. However, the role of HINT2 in the different stages of MASLD has not yet been explored, and the mechanism through which HINT2 regulates hepatic energy metabolism requires further investigation.

Sirtuin-3 (SIRT3) is involved in the regulation of acetylation of various intramitochondrial metabolic enzymes^9^. Anderson et al. reported that *Sirt3*-knockout mice exhibited a phenotype similar to *Hint2*-knockout mice, suggesting a possible functional linkage between HINT2 and SIRT3 (ref. ^10^). However, the mechanism by which HINT2 modifies the acetylation of mitochondrial proteins has not been elucidated.

In this study, we found that hepatic HINT2 was downregulated in patients with MASLD and in multiple mouse and cellular models of MASLD. In vitro, HINT2 inhibited free fatty acid (FFA)-induced lipid accumulation and impaired mitochondrial function in hepatocytes. Knockout of *Hint2* exacerbated diet-induced hepatic steatosis, inflammation, fibrosis and mitochondrial damage in mice, whereas overexpression of *Hint2* attenuated these effects. Mechanistically, HINT2 enhanced SIRT3 activity by upregulating solute carrier family 25 member 51 (SLC25A51) to facilitate NAD^+^ efflux, thereby attenuating mitochondrial injury and ameliorating MASLD. In MASLD, YTH N^6^-methyladenosine RNA binding protein 1 (YTHDF1) recognizes *Hint2* mRNA and regulates its stability, thereby altering the expression of HINT2.

## Materials and methods

### Animal models

C57BL/6 mice (male, ~8–10 weeks of age, ~18–22 g of weight) were purchased and housed in the Zhejiang Animal Center in temperature of 25 °C with a 12-h light/dark cycle and free access to water and food. *Hint2*-deficient (*Hint2*^*−*/*−*^) C57BL/6 mice were generated by Beijing View-Solid Biotechnology. The wild-type control mice were sibling littermates. Adeno-associated virus (AAV)-*Hint2* and control viruses were obtained from Hanbio. Wild-type control mice and *Hint2*^*−*/*−*^ mice aged ~8–10 weeks were fed a high-fat diet (HFD; 60% fat; D12492; Research Diets) for ~12–16 weeks, a methionine-and-choline-deficient (MCD) diet (A02082002BR; Research Diets) for 4 weeks or a western diet (WD; 42% kcal/fat diet, 41% sucrose, 1.25% Chol; TD.120528; Envigo) with sugar water (SW; 23.1 g/l d-fructose, 18.9 g/l d-glucose; Sigma) for 24 weeks. A standard chow diet (SCD; Research Diets) was used as a control. Interventions were done during the light cycle.

### Cell culture and treatments

The HepG2 cells, HEK293T cells and AML12 cells were purchased from the Chinese Academy of Sciences. All of the cells were incubated at 37 °C in 5% CO_2_. FFAs (oleic acid:palmitic acid 2:1; Sigma-Aldrich) were added at a concentration of 1 mM for 24 h to establish a cellular model of MASLD. siRNAs (RiboBio) and overexpression plasmids (Genechem) were transfected using Lipofectamine 3000 (Invitrogen). Lentivirus and control virus were produced by Hanbio.

### Human tissue samples

Liver tissues from patients with MASLD and healthy controls were obtained from individuals who underwent liver biopsy or liver resection for benign hepatic lesions at the First Affiliated Hospital, Zhejiang University School of Medicine.

### Study approval

Human liver tissues were obtained approved by the Ethics Committee of the First Affiliated Hospital, Zhejiang University School of Medicine (approval no. 2023-0422). All mouse experiments were approved by the appropriate ethics and the Animal Care and Use Committee of the First Affiliated Hospital, Zhejiang University School of Medicine (approval no. 2022-1603).

### Statistical analysis

All statistical analyses were performed using Prism 8 software (GraphPad). Quantitative data are displayed as mean ± s.d. Comparisons between groups were performed using two-tailed paired Student’s *t*-tests. One-way analysis of variance followed by Bonferroni analysis was performed for comparisons among three or more groups. *P* < 0.05 was considered to indicate statistical significance.

For further details regarding the materials and methods used, please refer to the Supplementary Information.

## Results

### HINT2 expression is decreased in patients and mice with MASLD and cellular models of MASLD

**Fig. 1:**
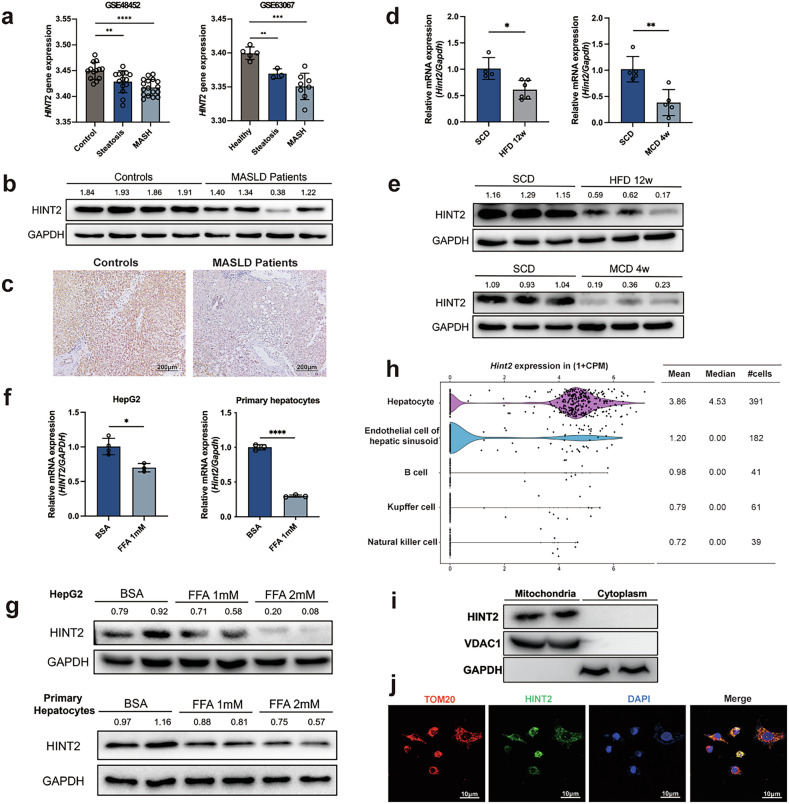
HINT2 expression is decreased in patients and mice with MASLD and cellular models of MASLD. **a** Effects of HINT2 on MASLD development revealed using two independent GEO datasets. **b**, **c** HINT2 expression in liver tissues of patients with MASLD and healthy controls. Scale bars, 200 μm. **d**, **e** mRNA expression level of *Hint2* (**d**) and protein level of HINT2 (**e**) in HFD-fed or MCD diet-fed mouse livers. **f**, **g** mRNA expression level of *HINT2* (**f**) and protein level of HINT2 (**g**) in FFA-stimulated HepG2 cells or primary hepatocytes. **h** Expression profiles of *Hint2* in mouse livers. **i** Protein expression of HINT2 in the mitochondria and cytoplasm of HepG2 cells. **j** Localization of TOM20 and HINT2 in HepG2 cells. Scale bars, 10 μm. Data are presented as mean ± s.d. *P < 0.05, **P < 0.01, ***P < 0.001, ****P < 0.0001 (Student’s *t*-test).

By analyzing Gene Expression Omnibus datasets, we found that hepatic *HINT2* expression was significantly lower in patients with MASLD than in control individuals (Fig. 1a). HINT2 protein expression was considerably lower in the liver tissues of patients with MASLD than in healthy controls (Fig. 1b, c). Similarly, compared with those of the SCD-fed controls, the mRNA and protein expression levels of HINT2 were significantly lower in the livers of mice fed a HFD for 12 weeks or a MCD diet for 4 weeks (Fig. 1d, e). mRNA and protein expression levels of HINT2 were also decreased in HepG2 cells and primary hepatocytes stimulated with different concentrations of FFA (Fig. 1f, g), and the decreased expression of HINT2 was further confirmed using an immunofluorescence assay (Supplementary Fig. 6b). Further single-cell transcriptomic analysis using the Tabula Muris database revealed that *Hint2* was preferentially expressed in hepatocytes among the five most abundant mouse liver cell types (Fig. 1h). Protein immunoblotting assays confirmed that HINT2 was expressed in the mitochondria of the hepatocytes (Fig. 1i), and we revealed the co-localization of HINT2 with TOM20 in HepG2 cells by cellular immunofluorescence (Fig. 1j), which also demonstrated the subcellular localization of HINT2. Moreover, HINT2 protein expression was markedly decreased in the mitochondria of FFA-stimulated hepatocytes and in the mitochondria of liver tissues from MASLD mice (Supplementary Fig. 6a). These results suggest a strong association between HINT2 and MASLD, indicating that HINT2 in the mitochondria may play an important role in MASLD.

### HINT2 regulates mitochondrial function and lipid accumulation in FFA-stimulated hepatocytes

**Fig. 2:**
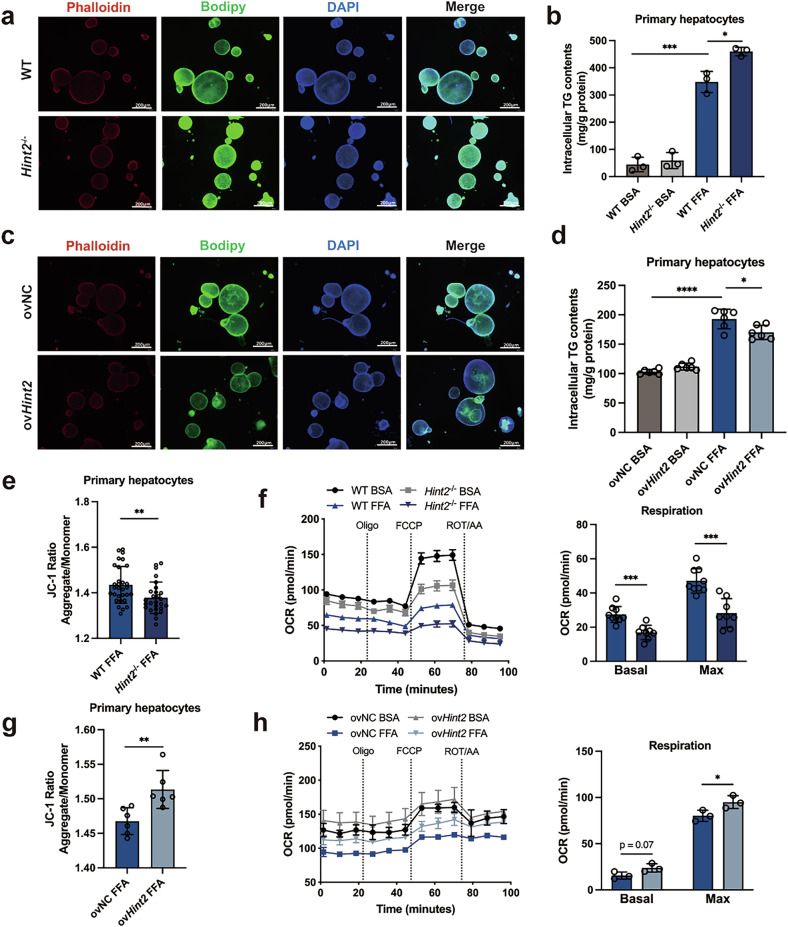
HINT2 regulates mitochondrial function and lipid accumulation in FFA-stimulated liver organoids and primary hepatocytes. Liver organoids or primary hepatocytes in which *Hint2* was knocked out or overexpressed were treated with 200 μM FFA for 48 h or 1 mM FFA for 24 h. **a** Lipid staining of *Hint2*^*−*/*−*^ liver organoids with BODIPY. Scale bars, 200 μm. **b** Intracellular TG contents of WT or *Hint2*^*−*/*−*^ primary hepatocytes. **c** Lipid staining of ovNC or ov*Hint2* liver organoids with BODIPY (ov, overexpression). Scale bars, 200 μm. **d** Intracellular TG contents of ovNC or ov*Hint2* primary hepatocytes. **e** Mitochondrial membrane potentials of WT or *Hint2*^*−*/*−*^ primary hepatocytes determined by the JC-1 ratios. **f** Mitochondrial respiratory functions of WT or *Hint2*^*−*/*−*^ primary hepatocytes determined by the OCR. The basal respiration and maximal respiration were compared. **g** JC-1 ratios of ovNC or ov*Hint2* primary hepatocytes. **h** OCR of ovNC or ov*Hint2* primary hepatocytes. The basal respiration and maximal respiration were compared. Scale bars: 50μm. Data are presented as mean ± SD. ^*^P < 0.05, ^**^P < 0.01, ^***^P < 0.001, ^****^P < 0.0001 (Student’s *t*-test).

The decreased expression of HINT2 in steatotic livers prompted us to explore whether HINT2 regulates MASLD development. We isolated and cultured liver organoids and primary hepatocytes from *Hint2*^*−*/*−*^ and wild-type mice and stained the lipids in organoids with BODIPY. *Hint2* deficiency significantly aggravated FFA-induced lipid accumulation in liver organoids (Fig. 2a) and increased the intracellular triglyceride (TG) content in primary hepatocytes (Fig. 2b). By contrast, the overexpression of *Hint2* significantly ameliorated FFA-induced lipid accumulation in liver organoids and increased intracellular TG concentration in primary hepatocytes (Fig. 2c, d).

We next determined whether HINT2 affects mitochondrial function. We found that *Hint2* deficiency significantly decreased the mitochondrial membrane potential and oxygen consumption rate (OCR), which included decreases in basal respiration and maximal respiration (Fig. 2e, f). By contrast, the overexpression of *Hint2* significantly improved mitochondrial function (Fig. 2g, h). We found similar results in HepG2 cells: knocking down *HINT2* expression in HepG2 cells exacerbated FFA-induced lipid accumulation and decreased the mitochondrial membrane potential and OCR (Supplementary Fig. 1a–d), whereas overexpressing of *HINT2* attenuated these alterations (Supplementary Fig. 1e–h). These findings demonstrate that HINT2 enhances mitochondrial activity and alleviates FFA-induced lipid accumulation in the hepatocytes.

### HINT2 attenuates hepatic steatosis and mitochondrial damage in MASL mice

**Fig. 3:**
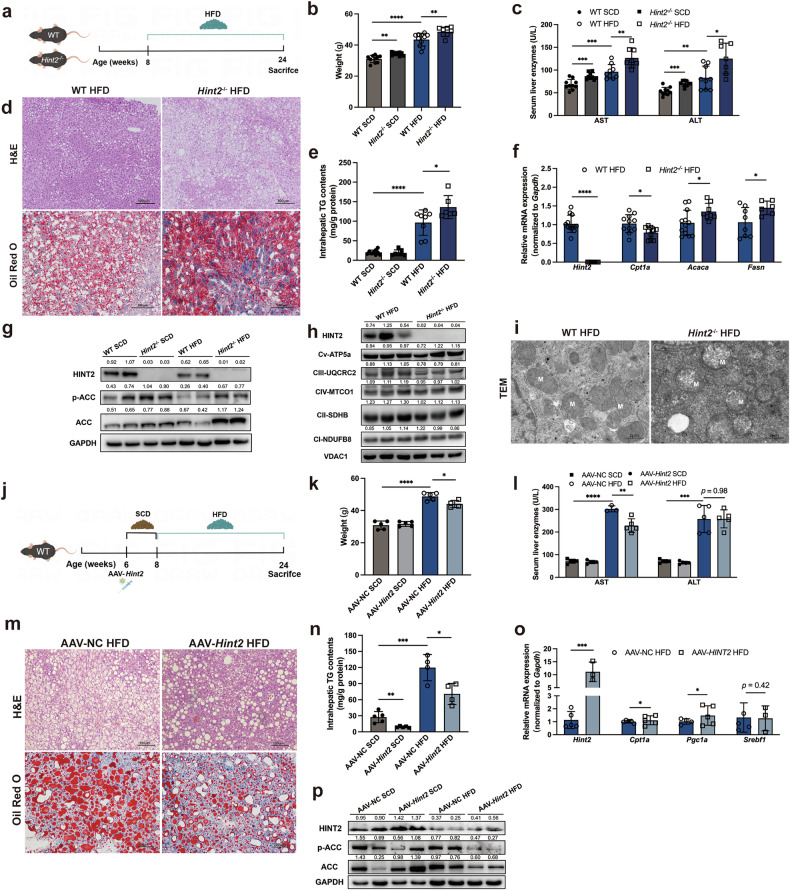
HINT2 attenuates hepatic steatosis and mitochondrial damage in MASL mice. **a** WT and *Hint2*^*−*/*−*^ mice were fed an HFD for 16 weeks. **b** Body weights of the mice. **c** Serum liver enzymes of the mice. **d** H&E and Oil Red O staining (scale bars, 100 μm) of mouse liver sections. **e** Intrahepatic TG contents of the mice. **f** qRT‒PCR analyses of the mRNA levels of genes related to lipid synthesis (*Acaca* and *Fasn*) and β-oxidation (*Cpt1a*) in mouse livers. **g** Western blot analyses of the protein levels of total and phosphorylated ACC in mouse livers. **h** Protein levels of OXPHOS complexes in mouse livers. **i** Transmission electron microscopy (TEM) (scale bars, 2 μm) of mouse liver sections. **j** WT mice were injected with *Hint2*-overexpressing AAV through the tail vein and fed an HFD for 16 weeks. **k** Body weights of the mice. **l** Serum liver enzymes of the mice. **m** H&E and Oil Red O staining of mouse liver sections. Scale bars, 100 μm. **n** Intrahepatic TG contents of mice. **o** qRT‒PCR analyses of the mRNA levels of genes related to lipid synthesis (*Srebf1*) and β-oxidation (*Cpt1a* and *Pgc1a*) in mouse livers. **p** Western blot analyses of the protein levels of total and phosphorylated ACC in mouse livers. Data are presented as mean ± s.d. *P < 0.05, **P < 0.01, ***P < 0.001, ****P < 0.0001 (Student’s *t*-test).

To investigate the function of HINT2 in MASLD, we induced metabolic dysfunction-associated steatotic liver (MASL), the early stage of MASLD, using ~12–16-week HFD feeding in mice. *Hint2*^*−*/*−*^ mice and their wild-type littermates were fed an HFD for 16 weeks (Fig. 3a). *Hint2*^*−*/*−*^ mice gained more body weight (Fig. 3b) and had higher aspartate transaminase (AST) and alanine aminotransferase (ALT) levels (Fig. 3c) than the wild-type controls after 16 weeks of HFD feeding. *Hint2* deficiency significantly exacerbated HFD-induced hepatic lipid deposition, as indicated by hematoxylin and eosin (H&E) and Oil Red O staining, and intrahepatic TG content (Fig. 3d, e). Consistent with the morphological findings, the relative mRNA expression levels of acetyl-CoA carboxylase alpha (*Acaca*) and fatty acid synthase (*Fasn*), two major genes involved in lipid synthesis, were higher in the livers of *Hint2*^*−*/*−*^ mice than in those of their wild-type littermates after HFD feeding, and the relative mRNA expression levels of carnitine palmitoyltransferase 1a (*Cpt1a*), which is involved in lipid oxidation, were lower than those in the latter mice (Fig. 3f). Compared with wild-type controls, HFD-fed *Hint2*^*−*/*−*^ mice also exhibited significantly greater hepatic protein expression of total and phosphorylated acetyl-CoA carboxylase (ACC) (Fig. 3g). In addition, *Hint2* deficiency inhibited the protein expression of mitochondrial respiratory chain complexes II, III and IV and exacerbated HFD-induced mitochondrial swelling, matrix granule loss and cristae breakage (Fig. 3h, i).

We next explored whether *Hint2* overexpression has the opposite effect. We overexpressed hepatic *Hint2* in wild-type mice via tail vein injection of *Hint2*-overexpressing AAV. Two weeks after virus injection, the mice were fed an SCD or an HFD for 16 weeks (Fig. 3j). The overexpression of *Hint2* reduced the HFD-induced weight gain and serum AST concentration in mice (Fig. 3k, l). Overexpression of *Hint2* also decreased the number of hepatic lipid droplets and intrahepatic TG content (Fig. 3m, n). Consistent with these findings, *Hint2* overexpression significantly upregulated *Cpt1a* and *Pgc1a* mRNA levels and downregulated the total and phosphorylated ACC protein levels in HFD-fed mice (Fig. 3o, p).

To further verify whether *Hint2* overexpression reverse *Hint2* deficiency-induced MASL exacerbation, we overexpressed hepatic *Hint2* in wild-type and *Hint2*^*−*/*−*^ mice, and fed the mice with an SCD or an HFD for 12 weeks two weeks after injection (Supplementary Fig. 2a). *Hint2* overexpression reduced the HFD-induced weight gain and increased serum liver enzyme levels and intrahepatic lipid accumulation in *Hint2*^*−*/*−*^ mice (Supplementary Fig. 2b–e). Moreover, overexpression of *Hint2* significantly reversed the changes in hepatic protein levels of total and phosphorylated ACC in *Hint2*^*−*/*−*^ mice (Supplementary Fig. 2f).

These findings provide clear evidence that HINT2 attenuates diet-induced MASL and hepatic mitochondrial impairment in mice.

###  HINT2 attenuates hepatic steatosis, inflammation, fibrosis and mitochondrial damage in MASH mice

**Fig. 4:**
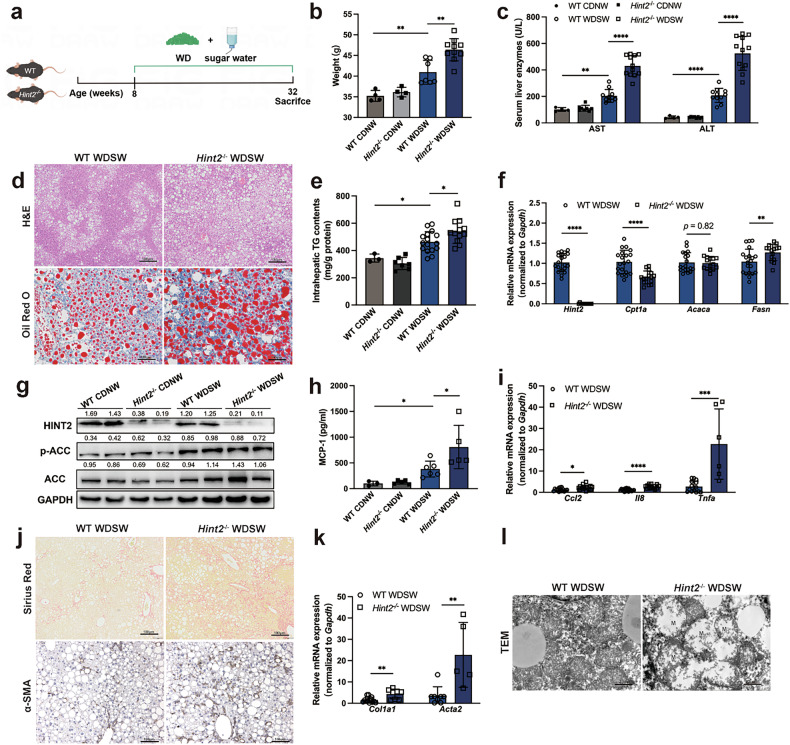
HINT2 attenuates hepatic steatosis, inflammation, fibrosis and mitochondrial damage in MASH mice. **a** WT and *Hint2*^*−*/*−*^ mice were fed a WDSW for 24 weeks. **b** Body weights of the mice. **c** Serum liver enzymes of the mice. **d** H&E and Oil Red O staining (scale bars, 100 μm) of mouse liver sections. **e** Intrahepatic TG contents of the mice. **f** mRNA levels of *Hint2*, *Cpt1a*, *Acaca* and *Fasn* in mouse livers. **g** Protein levels of total and phosphorylated ACC in mouse livers. **h** Serum MCP-1 concentrations of in mice. **i** mRNA levels of *Ccl2*, *Il8* and *Tnfa* in mouse livers. **j** Sirius Red and α-SMA staining (scale bars, 100 μm) of mouse liver sections. **k** mRNA levels of *Col1a1* and *Acta2* in mouse livers. **l** TEM (scale bars, 2 μm) of mouse liver sections. Data are presented as mean ± s.d. *P < 0.05, **P < 0.01, ***P < 0.001, ****P < 0.0001 (Student’s *t*-test).

To explore the regulatory effects of HINT2 in the next stages of MASLD, we induced a metabolic dysfunction-associated steatohepatitis (MASH) mouse model using WD + SW (Fig. 4a). *Hint2*^*−*/*−*^ mice gained more weight than wild-type mice after 24 weeks feeding with western diet and sugar water (WDSW) (Fig. 4b). *Hint2*^*−*/*−*^ mice also showed higher serum AST and ALT levels, larger increase in intrahepatic lipid deposition and TG content (Fig. 4c–e). The mRNA expression level of *Fasn* and the protein levels of total and phosphorylated ACC were significantly higher, and mRNA expression level of *Cpt1a* was lower in WDSW-fed *Hint2*^*−*/*−*^ mice than in the wild-type littermates (Fig. 4f, g). Furthermore, *Hint2* deficiency elevated the serum level of monocyte chemoattractant protein-1 (MCP-1) (Fig. 4h) and increased the mRNA expression of the proinflammatory factors *Ccl2*, *Il8* and *Tnfa* (Fig. 4i). To further evaluate the effects of HINT2 on hepatic fibrosis in MASH mice, Sirius Red staining and immunohistochemical staining were performed, which revealed a greater distribution of collagen fibers and alpha smooth muscle actin (α-SMA) proteins in the liver tissue of *Hint2*^*−*/*−*^ mice (Fig. 4j). Concordantly, *Hint2* deficiency also increased the expression of *Col1a1* and *Acta2* mRNAs in the livers (Fig. 4k). In addition, *Hint2* deficiency worsened the mitochondrial ultrastructural damage induced by WDSW (Fig. 4l).

We next verified the role of HINT2 in MASH using MCD feeding, an alternative dietary induction method for MASH (Supplementary Fig. 3a). We found that 4 weeks of MCD diet feeding markedly increased serum AST and ALT levels in wild-type mice, and these levels were further elevated in *Hint2*^*−*/*−*^ mice (Supplementary Fig. 3b). Compared with controls, *Hint2*^*−*/*−*^ mice also exhibited exacerbated hepatic lipid accumulation after MCD feeding (Supplementary Fig. 3c, d). The expression levels of lipid synthesis genes (*Acaca* and *Srebf1*) and total and phosphorylated ACC proteins were significantly upregulated in *Hint2*^*−*/*−*^ mice (Supplementary Fig. 3e, f). *Hint2* deficiency significantly elevated the expression of proinflammatory genes (*Il1b*, *Tnfa*, *Ccl2* and *Ccl5*), profibrotic genes (*Col1a1* and *Acta2*) and the serum level of MCP-1 in the livers of MCD-fed mice (Supplementary Fig. 3g–i). In addition, the mitochondria in the livers of *Hint2*^*−*/*−*^ mice exhibited more severe mitochondrial injuries (Supplementary Fig. 3j).

We also validate the findings using *Hint2*-overexpressing mice (Supplementary Fig. 3k). We found that overexpression of *Hint2* reduced serum liver enzymes and intrahepatic lipid deposition, inhibited lipid synthesis and promoted lipolysis in MCD-fed mice (Supplementary Fig. 3l–p). *Hint2* overexpression also reduced MCD-feeding induced liver inflammation and fibrosis (Supplementary Fig. 3q–s).

Taken together, these findings suggest that HINT2 attenuates hepatic steatosis, inflammation, fibrosis and mitochondrial damage in MASH mice and plays an important protective role in different stages of MASLD.

### HINT2 regulates protein deacetylation in mitochondria via SIRT3

**Fig. 5:**
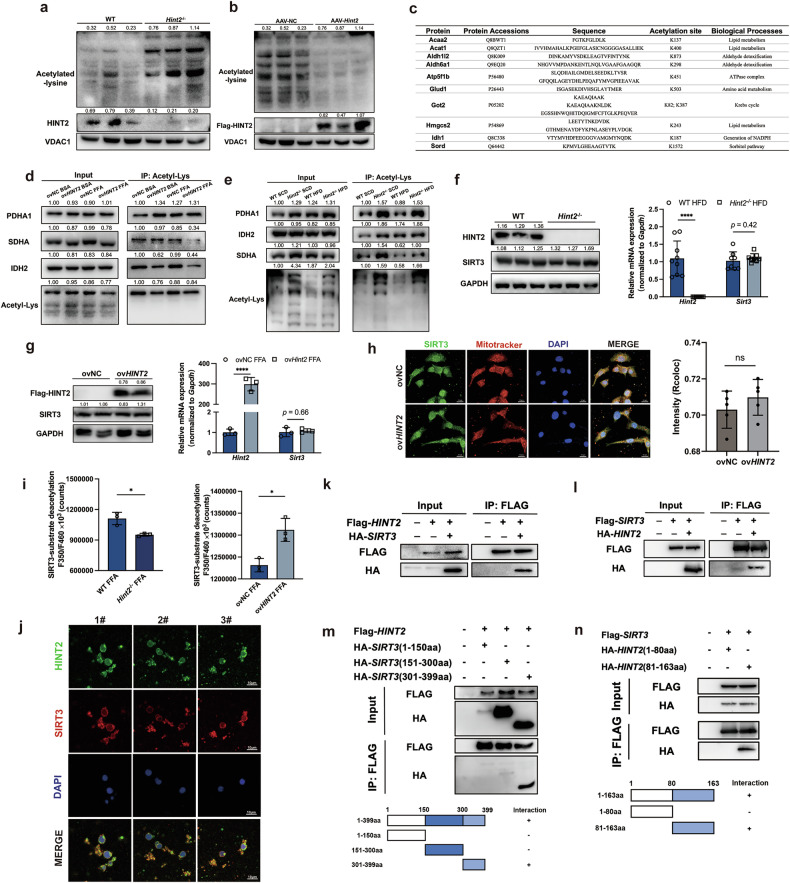
HINT2 regulates protein deacetylation via SIRT3. **a** The acetylation status of mitochondrial proteins in the livers of WT and *Hint2*^*−*/*−*^ mice. **b** Acetylation status of mitochondrial proteins in the livers of AAV-NC and AAV-*Hint2* mice. **c** Mass spectrometric analysis of proteins with altered acetylation modifications induced by HINT2. **d** Acetylation levels of PDHA1, SDHA and IDH2 in FFA-stimulated ovNC and ov*Hint2* HepG2 cells. **e** Acetylation levels of PDHA1, SDHA and IDH2 in the livers of HFD-fed WT and *Hint2*^*−*/*−*^ mice. **f** Protein and mRNA expression of SIRT3 in the livers of WT and *Hint2*^*−*/*−*^ mice. **g** Protein and mRNA expressions of SIRT3 in FFA-stimulated ovNC and ov*HINT2* HepG2 cells. **h** Co-localization of SIRT3 with mitochondria and its intensity. **i** SIRT3 activities in FFA-stimulated *Hint2*^*−*/*−*^ primary hepatocytes and ov*HINT2* HepG2 cells. **j** Representative immunofluorescent images of HINT2, SIRT3, DAPI and merge of all staining images of primary hepatocytes. Scale bars, 10 μm. **k**, **l** Co-IP and Western blot analyses of the interaction between HINT2 and SIRT3. Content of HA-*SIRT3* pulled down in HEK293T cells transfected with Flag-*HINT2* (**k**) Content of HA-*HINT2* pulled down in HEK293T cells transfected with Flag-*SIRT3* (**l**). in HEK293T cells transfected with Flag-*HINT2* or Flag-*SIRT3*. **m**, **n** The interaction domains of HINT2 and SIRT3 were explored using full-length and truncated SIRT3 (**m**) and HINT2 (**n**) expression constructs based on co-IP assays of HEK293T cells. Data are presented as mean ± s.d. *P < 0.05, ****P < 0.0001 (Student’s *t*-test).

However, the specific mechanism by which HINT2 regulates MASLD remains unclear. Martin et al. previously reported that *Hint2*^*−*/*−*^ mice exhibited a marked increase in mitochondrial protein acetylation^8^. Here, we confirmed that *Hint2* reduced the acetylation level of mouse mitochondrial proteins (Fig. 5a, b).

We then detected acetylated proteins in HepG2 cells by exogenous co-immunoprecipitation (co-IP) with acetylation profiling and found that very few proteins were pulled down by HINT2 and they were mainly cytoarchitectural proteins (Supplementary Fig. 6c). This suggests that HINT2 may not act as a deacetylase but is indirectly involved in the deacetylation reaction. We then compared the differentially accumulated acetylated hepatic proteins in wild-type and *Hint2*^*−*/*−*^ mice by mass spectrometry and found that *Hint2*^*−*/*−*^ mice exhibited higher acetylation levels of multiple enzymes involved in intramitochondrial metabolism than did wild-type mice (Fig. 5c). Differentially modified proteins were involved in lipid metabolism, amino acid metabolism, aldehyde detoxification and so on. Studies have shown that ACAT1, HMGCS2, GOT2 and GLUD1, which were included above, are substrates of SIRT3, the most predominant deacetylase in mitochondria^11–15^.

To further validate that HINT2-regulated proteins are associated with SIRT3, we performed co-IP assays and found that the overexpression of *HINT2* significantly decreased the acetylation levels of SDHA and IDH2 in HepG2 cells (Fig. 5d), whereas the knockout of *Hint2* increased the acetylation levels of PDHA1, IDH2 and SDHA in the livers of both SCD- and HFD-fed mice (Fig. 5e). Considering that the regulatory effect of HINT2 on the acetylation of liver mitochondrial proteins is probably related to SIRT3, and that SIRT3 is the most important deacetylase in mitochondria^15^, we next explored the role of HINT2 in regulating SIRT3. The protein and mRNA expression levels of SIRT3 were not altered by the knockout or overexpression of *Hint2* (Fig. 5f, g). We also confirmed that overexpression of HINT2 had no effect on the cellular localization of SIRT3 in mitochondria (Fig. 5h). Therefore, we considered the possibility that HINT2 may regulate SIRT3 enzymatic activity. The deacetylation activity of hepatic SIRT3 was significantly reduced in HFD-fed *Hint2*^*−*/*−*^ mice; conversely, the activity of SIRT3 was enhanced in FFA-treated HepG2 cells overexpressing *HINT2* (Fig. 5i).

We further explored whether HINT2 spatially regulates SIRT3 expression. Immunofluorescence staining of primary hepatocytes confirmed the co-localization of HINT2 with SIRT3, and co-IP assays indicated that HINT2 directly interacted with SIRT3 (Fig. 5j–l). Further experiments revealed that the 301–399 amino acid sequence (aa) of SIRT3 was responsible for direct interaction with HINT2 (Fig. 5m), whereas the 81–163 aa domain of HINT2 was found to be responsible for binding with SIRT3 (Fig. 5n). These results indicated that HINT2 altered the acetylation status of liver mitochondrial proteins by activating SIRT3.

### HINT2 ameliorates lipid metabolism and mitochondrial damage in vivo and in vitro by regulating SIRT3

**Fig. 6:**
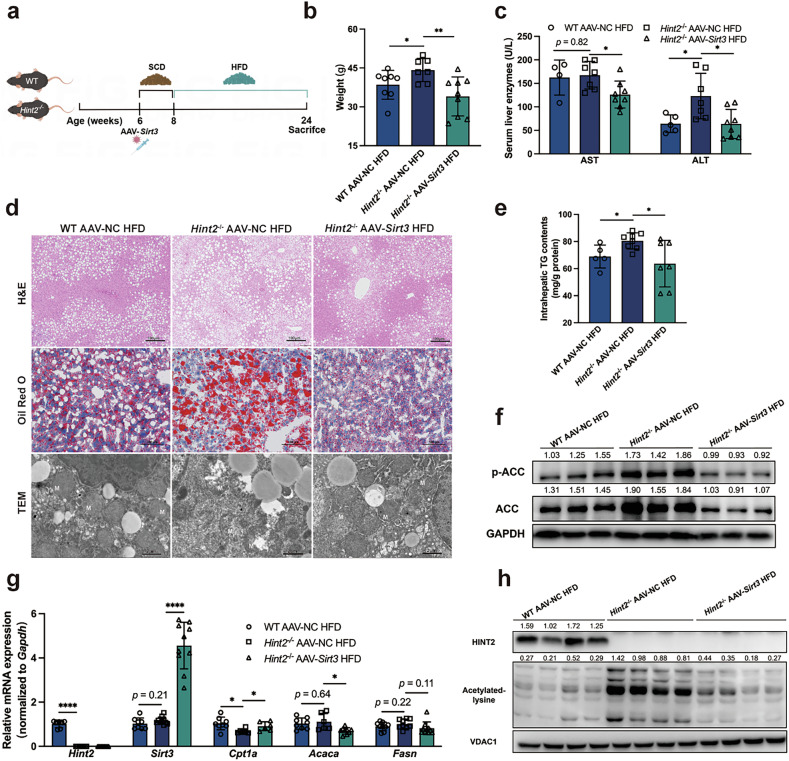
HINT2 ameliorates lipid metabolism and mitochondrial damage in vivo and in vitro by regulating SIRT3. **a** WT and *Hint2*^*−*/*−*^ mice were injected with *Sirt3*-overexpressing AAV through the tail vein and fed an HFD for 16 weeks. **b** Body weights of the mice. **c** Serum liver enzymes of the mice. **d** H&E, Oil Red O staining (scale bars, 100 μm) and TEM (scale bars, 2 μm) of mouse liver sections. **e** Intrahepatic TG contents of the mice. **f** Western blot analyses of the protein levels of total and phosphorylated ACC in mouse livers. **g** qRT‒PCR analyses of the mRNA levels of genes related to lipid synthesis (*Acaca* and *Fasn*) and β-oxidation (*Cpt1a*) in mouse livers. **h** Western blot analyses of mitochondrial protein acetylation status in mouse livers. Data are presented as mean ± s.d. *P < 0.05, **P < 0.01, ****P < 0.0001 (Student’s *t*-test).

We subsequently verified whether SIRT3 was responsible for the regulatory effects of HINT2 on MASLD. We generated *Hint2*^*−*/*−*^ mice with liver-specific *Sirt3* overexpression via tail vein injection of AAV-*Sirt3* and fed them a SCD or an HFD for 16 weeks (Fig. 6a). *Sirt3* overexpression effectively decreased body weight gain, serum AST and ALT levels and intrahepatic TG content, ameliorated hepatic steatosis and reversed mitochondrial structural damage (Fig. 6b–e) in HFD-fed *Hint2*^*−*/*−*^ mice. Furthermore, *Sirt3* overexpression decreased total and phosphorylated ACC protein expression (Fig. 6f), reduced *Acaca* mRNA expression and upregulated *Cpt1a* mRNA expression (Fig. 6g) in HFD-fed *Hint2*^*−*/*−*^ mice. Hyperacetylation of mitochondrial proteins induced by *Hint2* deficiency was also ameliorated by *Sirt3* overexpression (Fig. 6h).

We further explored whether SIRT3 mediated the effect of HINT2 on MASLD in vitro. We knocked down *SIRT3* by infection with lentivirus-sh*SIRT3* and found that the reduction in *SIRT3* expression significantly aggravated FFA-induced lipid accumulation but abolished the ameliorative effects of *HINT2* overexpression in HepG2 cells (Supplementary Fig. 4a, b). *SIRT3* knockdown also markedly decreased mitochondrial membrane potential and OCR in FFA-stimulated HepG2 cells, whereas *HINT2* overexpression had no restorative effect on these cells (Supplementary Fig. 4c, d). By contrast, the overexpression of *Sirt3* significantly ameliorated FFA-induced lipid accumulation in liver organoids and primary hepatocytes from *Hint2*^*−*/*−*^ mice (Supplementary Fig. 4e, f). *Sirt3* overexpression also restored mitochondrial membrane potential and respiratory function in *Hint2*^*−*/*−*^ primary hepatocytes (Supplementary Fig. 4g, h).

These findings suggest that HINT2 ameliorates MASLD through a SIRT3-dependent mechanism, which associated with improved mitochondrial damage.

### HINT2 regulates SIRT3 activity by modifying the NAD^+^ concentration and thus affects MASLD

However, the mechanisms by which HINT2 regulates SIRT3, thereby ameliorating MASLD, remain unclear. SIRT3 is an NAD^+^-dependent deacetylase that requires NAD^+^ as a co-substrate^16,17^. Thus, we explored whether HINT2 activates SIRT3 by altering the abundance of NAD^+^ in the mitochondria. In vitro, NAD^+^ concentration and SIRT3 deacetylation activity were significantly decreased in the mitochondria of FFA-stimulated primary hepatocytes and HepG2 cells (Supplementary Fig. 6d,e). Knockout of *Hint2* decreased NAD^+^ concentration in FFA-stimulated primary hepatocytes, whereas overexpression of *HINT2* significantly elevated NAD^+^ levels in FFA-stimulated HepG2 cells (Supplementary Fig. 5a). In vivo, *Hint2* overexpression significantly reversed the HFD-induced reduction in mitochondrial NAD^+^ concentration in the mouse liver, whereas *Hint2* deletion further reduced mitochondrial NAD^+^ concentration (Supplementary Fig. 6f, g). These findings suggest that HINT2 contributes to increasing mitochondrial NAD^+^ concentration, which is probably the mechanism through which HINT2 activates SIRT3.

To further verify this speculation, we supplemented NAD^+^ with mononucleotide (NMN), a precursor and booster of NAD^+^ (ref. ^18^). Treatment with NMN restored the reduced NAD^+^ concentration and SIRT3 deacetylation activity in FFA-stimulated *Hint2*^*−*/*−*^ derived primary hepatocytes (Supplementary Fig. 5b). Accordingly, NMN administration restored the protein hyperacetylation status of primary hepatocytes in *Hint2*^*−*/*−*^ mice (Supplementary Fig. 5c). To further investigate the role of HINT2 in promoting increases in NAD^+^ concentration in MASLD, *Hint2*^*−*/*−*^ mice and wild-type controls were fed an HFD for 12 weeks and received intraperitoneal injections of 500 mg/kg NMN or saline every 2 days for 4 weeks beginning at week 9 (Supplementary Fig. 5d). Intraperitoneal administration of NMN greatly replenished intrahepatic mitochondrial NAD^+^ concentration and enhanced SIRT3 activity in mice (Supplementary Fig. 5e). NMN decreased weight gain, serum AST and ALT levels and intrahepatic TG content and ameliorated hepatic steatosis and mitochondrial structural injuries in HFD-fed *Hint2*^*−*/*−*^ mice (Supplementary Fig. 5f–i). NMN supplementation also significantly increased *Cpt1a* mRNA levels, decreased *Fasn* and *Acaca* mRNA levels and decreased total and phosphorylated ACC protein levels in HFD-fed *Hint2*^*−*/*−*^ mice (Supplementary Fig. 5j, k). These findings suggest that HINT2 promotes an increase in NAD^+^ levels to activate SIRT3, which provides protection against MASLD.

### HINT2 promotes NAD^+^ mitochondrial influx by facilitating mitochondrial SLC25A51 enrichment

We further investigated how HINT2 increased mitochondrial NAD^+^ concentration. Nicotinamide mononucleotide adenylyl transferase 3 (NMNAT3) is a key enzyme that catalyzes NAD^+^ biosynthesis and is localized in the mitochondrial matrix^19,20^. We assessed the effect of HINT2 on NMNAT3 protein expression. Neither overexpression nor deletion of *Hint2* in vivo affected the expression of NMNAT3 (Supplementary Fig. 6h, i).

**Fig. 7:**
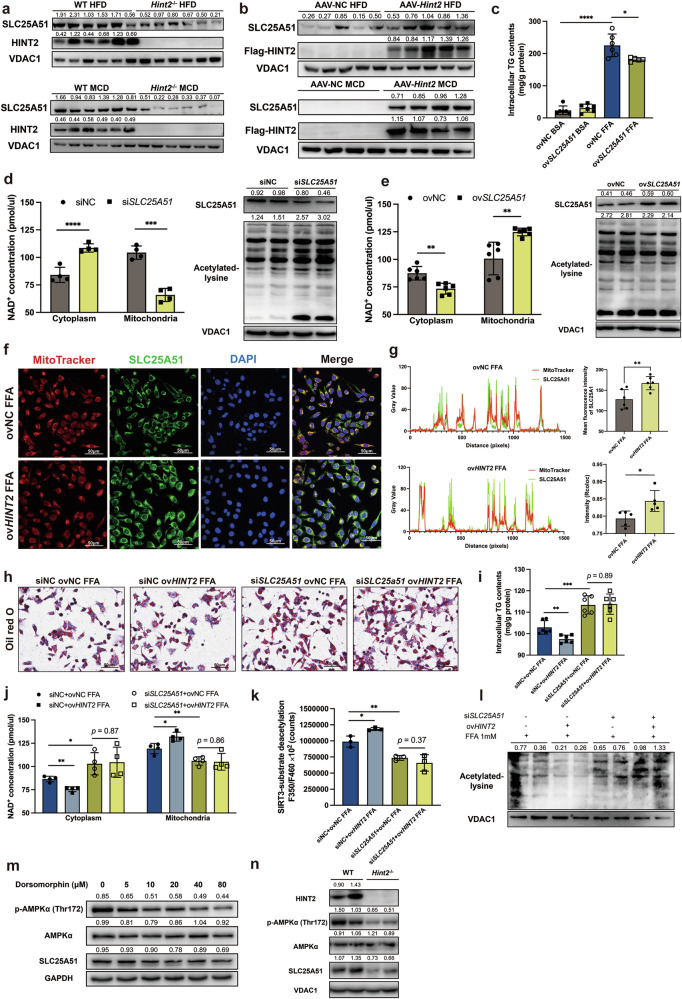
HINT2 facilitates NAD^+^ mitochondrial influx through SLC25A51. **a** The mitochondrial protein expression of SLC25A51 in the livers of HFD or MCD-fed WT and *Hint2*^*−*/*−*^ mice. **b** The mitochondrial protein expression of SLC25A51 in the livers of HFD or MCD-fed AAV-NC and AAV-*Hint2* mice. **c** Intracellular TG contents of FFA-stimulated *SLC25A51*-overexpressing HepG2 cells. **d** NAD^+^ concentrations in cytoplasm and mitochondria, and acetylation status of mitochondrial proteins in *SLC25A51*-silenced HepG2 cells. **e** NAD^+^ concentrations in cytoplasm and mitochondria, and acetylation status of mitochondrial proteins in ov*SLC25A51* HepG2 cells. **f** The localization of SLC25A51 in mitochondria of ov*HINT2* HepG2 cells. Scale bars, 50 μm. **g** The fluorescence intensity of SLC25A51 and MitoTracker on the diagonal of the merge image of **f** and the fluorescence intensity of SLC25A51 in **f**. **h** Oil Red O staining of si*SLC25A51* ov*HINT2* HepG2 cells. Scale bars, 50 μm. **i** Intracellular TG contents of si*SLC25A51* ov*HINT2* HepG2 cells. **j** NAD^+^ concentrations in cytoplasm and mitochondria of si*SLC25A51* ov*HINT2* HepG2 cells. **k** SIRT3 activities of si*SLC25A51* ov*HINT2* HepG2 cells. **l** The acetylation status of mitochondrial proteins in si*SLC25A51* ov*HINT2* HepG2 cells. **m** The protein expression of p-AMPKa and AMPK in dorsomorphin-treated HepG2 cells. **n** The protein expression of p-AMPKa and AMPK in mitochondria of *Hint2*^*−*/*−*^ mouse liver. Data are presented as mean ± s.d. *P < 0.05, **P < 0.01, ***P < 0.001, ****P < 0.0001 (Student’s *t*-test).

Solute carrier family 25 member 51 (SLC25A51), also known as mitochondrial carrier triple repeat 1 (MCART1), encoded by *slc25a51*, is recently identified as a mammalian mitochondrial NAD^+^ transporter that is essential for maintaining the homeostasis of the mitochondrial NAD^+^ pool^21,22^. We found that knockout of *Hint2* significantly decreased the mitochondrial protein expression of SLC25A51 in the livers of MASLD model mice, whereas overexpression of *Hint2* significantly elevated the expression of SLC25A51 (Fig. 7a, b).

We evaluated the possible effects of SLC25A51 on MASLD and found that overexpression of *SLC25A51* reduced the FFA-induced increase in intracellular TG concentration in HepG2 cells (Fig. 7c). Knockdown of *SLC25A51* elevated cytoplasmic NAD^+^ concentration, decreased mitochondrial NAD^+^ concentration and upregulated mitochondrial protein acetylation in HepG2 cells, whereas overexpression of *SLC25A51* decreased cytoplasmic NAD^+^ concentration, elevated mitochondrial NAD^+^ concentration and further downregulated mitochondrial protein acetylation in HepG2 cells (Fig. 7d, e). These results verified the ability of SLC25A51 in hepatocytes to promote mitochondrial NAD^+^ influx, regulate mitochondrial protein acetylation status and reduce lipid accumulation.

To explore how HINT2 acts on SLC25A51 in MASLD, we investigated the effect of HINT2 on the distribution of SLC25A51 in mitochondria and found that overexpression of *Hint2* promoted the expression of SLC25A51 in mitochondria and enhanced its co-localization with mitochondria in FFA-stimulated HepG2 cells (Fig. 7f, g). Next, we verified whether SLC25A51 mediates the regulatory effects of HINT2 in MASLD. Silencing of *SLC25A51* significantly aggravated FFA-induced lipid accumulation in HepG2 cells but abolished the ameliorative effects of *HINT2* overexpression (Fig. 7h, i). Moreover, knockdown of *SLC25A51* decreased NAD^+^ influx and SIRT3 activity and upregulated mitochondrial protein acetylation in HepG2 cells, whereas overexpression of *HINT2* had no restorative effect on NAD^+^ influx, SIRT3 activity or mitochondrial protein acetylation status in *SLC25A51*-silenced cells (Fig. 7j–l). These results suggest that HINT2 enhances SIRT3 activity by promoting SLC25A51 enrichment, thereby facilitating NAD^+^ influx into mitochondria.

Next, we explored the mechanism of HINT2 on promoting SLC25A51 expression in mitochondria. We performed co-IP and found that HINT2 and SLC25A51 did not bind directly (Supplementary Fig. 6j). Based on the fact that AMPK activity has an important role in mitochondrial biogenesis, we inhibited AMPK activity with dorsomorphin in HepG2 cells. We found that SLC25A51 expression was downregulated accompanied by AMPK inactivation (Fig. 7m). We further found that *HINT2* deficiency significantly inhibited AMPK activity in mitochondria (Fig. 7n). These findings suggest that HINT2 may contribute to the reduction of SLC25A51 by inhibiting AMPK activity in mitochondria.

### Downregulation of YTHDF1 in MASLD reduces the mRNA stability of *Hint2*

**Fig. 8:**
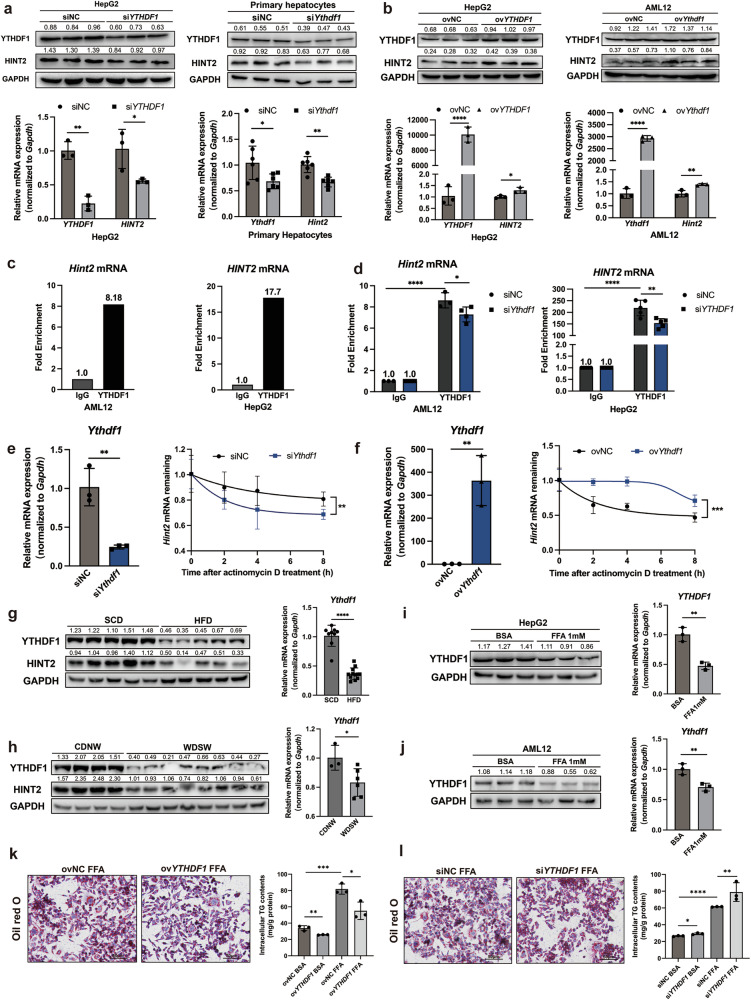
Downregulation of YTHDF1 in MASLD reduces the mRNA stability of *Hint2.* **a** Protein and mRNA expression of HINT2 in si*YTHDF1* HepG2 cells and si*Ythdf1* primary hepatocytes. **b** Protein and mRNA expression of HINT2 in ov*YTHDF1* HepG2 cells and ov*Ythdf1* AML12 cells. **c** The strength of *Hint2* mRNA binding to YTHDF1 in hepatocytes. **d** The strength of *Hint2* mRNA binding to YTHDF1 in *Ythdf1*-silenced hepatocytes. **e** *Hint2* mRNA stabilities after actinomycin D treatment in *Ythdf1*-silenced AML12 cells. **f** *Hint2* mRNA stabilities after actinomycin D treatment in *Ythdf1*-overexpressing AML12 cells. **g**, **h** Protein and mRNA expression of YTHDF1 in the livers of HFD- (**g**) and WDSW- (**h**) fed mice. **i**, **j** Protein and mRNA expression of YTHDF1 in FFA-stimulated HepG2 cells (**i**) and AML12 cells (**j**). **k** Oil Red staining and intracellular TG contents of HepG2 cells overexpressing *YTHDF1* under FFA stimulation. **l** Oil Red staining and intracellular TG contents of HepG2 cells silencing *YTHDF1* under FFA stimulation. Data are presented as mean ± s.d. *P < 0.05, **P < 0.01, ***P < 0.001, ****P < 0.0001 (Student’s *t*-test).

However, the mechanism underlying decreased HINT2 expression in MASLD remains unclear. HINT2 expression has been reported to be positively regulated by YTHDF1-mediated m^6^A modification in ocular melanoma^23^. This led us to consider whether the reduction in HINT2 expression in MASLD was associated with the modification of *Hint2* mRNA and whether it was associated with YTHDF1. We thus explored the effect of YTHDF1 on HINT2 expression. The mRNA and protein expression levels of HINT2 were significantly reduced after *Ythdf1* was silenced in hepatocytes and were significantly increased after *Ythdf1* overexpression (Fig. 8a, b), indicating that YTHDF1 regulates the expression of HINT2 in hepatocytes.

The results of the RIP assays suggested that YTHDF1 binds *Hint2* mRNA in hepatocytes and that this binding is significantly weakened after *Ythdf1* is silenced (Fig. 8c, d). To explore how YTHDF1 acts on *Hint2* mRNA, we assessed the effect of YTHDF1 on the stability of *Hint2* mRNA. *Hint2* mRNA degradation was faster in *Ythdf1*-silenced AML12 cells than in normal control cells; conversely, *Hint2* mRNA degradation was suppressed after *Ythdf1* was overexpressed (Fig. 8e, f). These results indicated that YTHDF1 is essential for maintaining the stability of *Hint2* mRNA.

To investigate whether the decrease in HINT2 in MASLD is correlated with YTHDF1, we detected changes in the expression of YTHDF1 in the livers of MASLD model mice and hepatocytes. The mRNA and protein expression levels of YTHDF1 in the livers of mice fed HFD for 16 weeks and mice fed WDSW for 24 weeks were significantly lower than those in the livers of control mice (Fig. 8g, h). Moreover, YTHDF1 expression was significantly reduced in the FFA-stimulated HepG2 and AML12 cells (Fig. 8i, j). Overexpression of *YTHDF1* in HepG2 cells alleviated FFA-induced lipid accumulation, while silencing *YTHDF1* aggravated cell lipid accumulation (Fig. 8k, l). These results suggest that YTHDF1 expression is decreased in MASLD and thus inhibits HINT2 in MASLD by affecting *Hint2* mRNA stability.

## Discussion

In this study, we explored the role of the mitochondrial protein HINT2 in MASLD pathogenesis. Previous studies have shown that HINT2 plays a positive role in regulating hepatocyte energy metabolism and that *Hint2*^*−*/*−*^ mice are more likely to exhibit increased hepatic steatosis^8,24,25^. These findings suggested that *Hint2*^*−*/*−*^ mice are more susceptible to MASLD. However, whether HINT2 expression is associated with different stages of MASLD and whether *Hint2* overexpression ameliorates MASLD remains unclear. In this study, we confirmed that HINT2 expression is decreased in patients with simple fatty liver and MASH using Gene Expression Omnibus data analysis and verified the reduction of HINT2 in patients with MASLD. Based on findings in vitro and in vivo, we identified a correlation between HINT2 and MASLD. Three mouse models were established to simulate MASL and MASH, by which we further explored the etiological relationship between the reduction in HINT2 and the development of MASLD at different stages of the disease.

Protein lysine acetylation is an important posttranslational modification of proteins that is an important link between liver metabolism and mitochondrial function^26,27^. Recent studies have shown that the acetylation status of certain metabolic enzymes in the mitochondria is associated with MASLD^28–30^. It has been reported that HINT2 regulates the deacetylation of mitochondrial proteins^8,24,31^. We also validated a similar function of HINT2 in mouse livers.

Protein acetylation is regulated mainly by a family of NAD^+^-dependent protein deacetylases known as sirtuins^32^. Of the sirtuins, only mice lacking *Sirt3* showed increased levels of altered mitochondrial protein acetylation, suggesting that SIRT3 is the major mitochondrial protein deacetylase^15^. *Sirt3*-knockout mice reportedly develop fatty liver, glucose intolerance and decreased respiratory function^30,33^, and the phenotype of these mice is similar to that of *Hint2*^*−*/*−*^ mice. Indeed, we found that the protein acetylation modification of HINT2 is associated with SIRT3. Therefore, we explored the potential of HINT2 to regulate SIRT3 expression. Our results were consistent with those reported by Fan et al., who found that HINT2 did not affect the protein expression of SIRT3 (ref. ^31^); however, we found that HINT2 enhanced the deacetylation activity of SIRT3. We confirmed that SIRT3 is an essential component mediating the protective effect of HINT2 against MASLD.

NAD^+^ is an important oxidative reducer of energy metabolism and a substrate for a range of enzymes^34,35^. SIRT3 mediates the deacetylation reaction that requires NAD^+^ as a cofactor^16,17^. The enzymatic activity of SIRT3 is correlated with the NAD^+^/NADH ratio or absolute levels of NAD^+^, NADH and NAM (the NAD^+^ pool)^36–38^. Thus, we hypothesized that HINT2 alters SIRT3 activity by regulating NAD^+^ pool homeostasis. Consistent with our expectation, HINT2 positively regulated mitochondrial NAD^+^ concentration, promoted the enzymatic activity of SIRT3 and decreased the acetylation level of mitochondrial proteins. Fan et al. reported that *Hint2* overexpression complemented mitochondrial NAD^+^ levels in cardiomyocytes^31^, which is consistent with our results. In addition, these researchers have predicted the potential binding of HINT2 to NMN. NMN has been found to improve hepatic mitochondrial function and decrease oxidative stress in preclinical MASLD models^35^. We reversed the changes in NAD^+^ levels in *Hint2*^*−*/*−*^ livers and hepatocytes by supplementing the substrate for NAD^+^ synthesis with exogenous NMN, which restored SIRT3 activity and attenuated HFD-induced MASLD. These results indicate a novel mechanism by which HINT2 regulates SIRT3 activity via the NAD^+^ pool, thereby ameliorating MASLD.

SLC25A51, also known as MCART1, a mammalian mitochondrial NAD^+^ transporter that has gained increasing attention in the last three years, is capable of transporting NAD^+^ from the cytoplasm to the mitochondria^21,39^. It has been reported that inhibition of *Slc25a51* expression in hepatocytes decreases mitochondrial NAD^+^ levels and SIRT3 activity. Mice with reduced hepatic *Slc25a51* expression exhibit hepatic steatosis and hypertriglyceridemia after fasting^40^. First, we found that HINT2 positively regulates SLC25A51 expression and that SLC25A51 has an inhibitory effect on TG accumulation in hepatocytes. These findings led us to focus on the regulatory effect of HINT2 on NAD^+^ homeostasis and biological function of SLC25A51. We tentatively found that HINT2 may promote SLC25A51 enrichment in mitochondria and further found that HINT2 promotes NAD^+^ influx through SLC25A51, thereby facilitating the deacetylation activity of SIRT3 and attenuating lipid deposition in hepatocytes.

NAD^+^ could be imported into mitochondria via SLC25A51 and reversibly cleaved to nicotinamide (NAM) mononucleotide and ATP in the presence of NMNAT3, thereby maintaining an additional ‘virtual’ NAD^+^ pool. This mechanism allows the maintenance of overall NAD^+^ levels by subcellular-to-subcellular NAD^+^ transfer when NAD^+^ is depleted excessively^41^. However, the salvage pathway of NAD^+^ is dominant in the maintenance of intracellular NAD^+^ homeostasis. When levels of NAD^+^ decline, this pathway can be enhanced by reconverting metabolites of NAD^+^ (for example, NAM) to NAD^+^ (ref. ^9^). Thus, under normal conditions, NAD^+^ transport mediated by HINT2 does not result in significant changes in mitochondrial NAD^+^ contents. When hepatocytes are fat-loaded, NAD^+^ metabolism is affected by abnormal mitochondrial function, and NAD^+^ contents in mitochondria are decreased^41^. We observed similar results in this study, finding that the mitochondrial NAD^+^ concentration in FFA-treated hepatocytes was significantly lower than in those treated with bovine serum albumin (BSA), and the liver mitochondrial NAD^+^ concentration in HFD-fed mice was significantly lower than in control mice (Supplementary Fig. 6d–g). In that condition, the effect of *Hint2* intervention on NAD^+^ contents may have been more dramatic, and so it was shown that HINT2-mediated mitochondrial NAD^+^ influx occurred only in the presence of excess fat. This speculation requires further experimental verification.

The YTH family is an m^6^A reader that regulates the translation of m^6^A-modified transcripts. It has been reported that YTHDF1 recognizes the m^6^A modification of HINT2 in malignant ocular melanoma and thus regulates its translation^23^. Therefore, we explored the effect of YTHDF1 on HINT2 expression. Our results showed that *Ythdf1* intervention altered HINT2 expression and that YTHDF1 bound to and improved the stability of *Hint2* mRNA. This suggests that m^6^A modification of *Hint2* mRNA is recognized by YTHDF1 and that the low expression of YTHDF1 in MASLD reduces its stability and decreases HINT2 expression.

The mechanism by which HINT2 functions as a deacetylator via SIRT3 to improve mitochondrial function and MASLD remains unclear. In this study, we found that HINT2 regulates the acetylation modification of several lipid metabolism-related proteins in the liver by acetylated protein profiling. Acetyl-CoA acyltransferase 2 (ACAA2) plays a role in the degradation of isoleucine and is involved in the regulation of mitochondrial fatty acid oxidation^42^. Deacetylation of ACAA2 by SIRT3 has been reported not only to affect its activity but also to be critical for the regulation of mitochondrial morphology and function^43^. Acetyl-CoA acetyltransferase 1 (ACAT1) is involved in the formation and catabolism of ketones, isoleucines and fatty acids, and its activity is essential for the maintenance of mitochondrial acetyl-CoA levels^44^. ACAT1 is regulated by SIRT3, which is reversibly inhibited by acetylation at specific lysine residues, thereby regulating mitochondrial metabolism^11^. 3-Hydroxy-3-methylglutaryl-CoA synthase 2 (HMGCS2) is also regulated by SIRT3, and its activity is reduced in the presence of acetylation of HMGCS2, leading to reduced ketone body production^45^. Tacrolimus has been reported to decrease HMGCS2 expression, which in turn decreases ketone body production, leading to lipid accumulation in hepatocytes^46^.

In this study, we found that deletion of *Hint2* reduced hepatic mitochondrial NAD^+^ influx via SLC25A51, in which case the overexpression of *Sirt3* was still functional. As previously described, the NAD^+^ salvage pathway was activated under energy burdens^47^. Although the deletion of *Hint2* inhibited the influx of NAD^+^, the enhancement of salvage pathway in the conditions of excess fat might enable supplemented NAD^+^ to activate overexpressed *Sirt3*. Besides, previous studies reported that overexpression of *Sirt3* could also promote substrate deacetylation in the absence of additional NAD^+^ supplementation^29,48,49^. Those reasons may explain why overexpression of *Sirt3* could attenuate HFD-induced MASLD in *Hint2*^*−*/*−*^ mice. Further studies are needed to clarify this hypothesis.

We tentatively explored the possible regulatory mechanisms of HINT2 on SLC25A51. We found that the activity of AMPK in hepatocytes regulated SLC25A51 expression and that the deficiency of *Hint2* inhibited AMPK activity in mitochondria of hepatocytes. This suggests that HINT2 may regulate the expression of SLC25A51 by modulating mitochondrial AMPK activity. However, the role of AMPK in the regulation of SLC25A51 by HINT2 remains unclear and requires further validation. Further studies of possible regulatory mechanisms of HINT2 on SLC25A51 are also warranted.

Our research suggested that HINT2 may be a potential therapeutic target for MASLD; thus, targeting HINT2 in humans needs to be further explored. A study in acute pancreatitis found that the drug Emodin targeted HINT2 to promote mitochondrial oxidative phosphorylation (OXPHOS), attenuating inflammatory responses and cell necrosis^50^. This suggests that Emodin may be a potential pharmacologic agent for enhancing HINT2 function. In addition, it is also possible to increase HINT2 expression by importing the HINT2 gene into hepatocytes using gene editing techniques such as CRISPR–Cas9. However, there are relative side effects and challenges associated with targeting HINT2. HINT2 is expressed in tissues such as the liver, pancreas and adrenal glands, and targeted therapies may have nonspecific effects on these tissues. Gene therapies targeting HINT2 that are not consistently expressed in the liver may diminish therapeutic efficacy in MASLD.

Our study had several limitations. First, we used global *Hint2*-knockout mice in this study. Although HINT2 is expressed at high levels in the liver, systemic changes by globally knocking out *Hint2* may affect the observed effects rather than using liver-specific *Hint2*-knockout mice. Second, our *Hint2* loss-of-gain approaches involve systemic or whole-cell approaches and do not specifically target the mitochondria. An efficient method for controlling mitochondria needs to be identified. Third, whether there are alternative ways in which HINT2 is regulated in MASLD remains to be explored. Finally, the key downstream proteins deacetylated by SIRT3 that affect MASLD development have not been identified.

In summary, this study provides evidence for the protective effect of HINT2 against MASLD. Mechanistically, the protective effect of HINT2 on MASLD was mediated by the activation of SIRT3 deacetylation through the promotion of NAD^+^ mitochondrial efflux by SLC25A51. The low expression of HINT2 in MASLD is due to the YTHDF1-mediated regulation. Therefore, the specific targeting of HINT2 may be an effective clinical strategy for the treatment of MASLD.

## Supplementary information

Supplementary Information
