# Supplementary material for: Histidine triad nucleotide-binding protein 2 attenuates metabolic dysfunction-associated steatotic liver disease through NAD+-dependent sirtuin-3 activation
Source: Exp Mol Med. 2025 May 1;57(5):990–1004. doi: 10.1038/s12276-025-01445-w (PMC12130462; doi:10.1038/s12276-025-01445-w)
Supplement: Supplementary file 1 — Supplementary Information [file 12276_2025_1445_MOESM1_ESM.pdf]

Histidine triad nucleotide-binding protein 2 attenuates metabolic dysfunction-associated steatotic liver disease through NAD^+-dependent sirtuin-3 activation

Qinqiu Wang[1]; Yanjun Guo[1]; Shenghui Chen[1]; Zhening Liu[1]; Xinyu Wang[1]; Hangkai Huang[1]; Qi-en Shen[1]; Ling Yang[1]; Meng Li[1]; Youming Li[1]; Chaohui Yu[1]; Chengfu Xu[1*]

[1]Department of Gastroenterology, the First Affiliated Hospital, Zhejiang University School of Medicine, Hangzhou 310003, China

***Corresponding author:** Chengfu Xu, MD, Department of Gastroenterology, the First Affiliated Hospital, Zhejiang University School of Medicine, 79 Qingchun Road, Hangzhou 310003, China, Tel: 0086-571-87236863, E-mail: xiaofu@zju.edu.cn

Supplementary materials and methods

Histological analysis

For immunohistochemistry, tissue slides were incubated with primary antibodies against HINT2 (LS-C404041; LifeSpan, Shirley, MA) and with secondary antibodies (ZSGB-BIO, Beijing, China). The liver tissues were rapidly frozen in liquid nitrogen, sectioned by freezing and then stained with Oil red O using a kit (D027; njjcbio, Nanjing, China). The preparation of electron microscopy samples and image acquisition were performed by Servicebio Biotechnology Company (Wuhan, China).

Isolation of liver organoids

Mouse livers were dissociated with Collagenase Type IV (17104019, Gibco) and Dispase (07923, STEMCELL) into hepatic ducts in a 37°C water bath. The suspension was successively filtered through 70μm and 37μm cell sieve, and after centrifugation, the precipitate was mixed with matrix gel (356231, Corning) and seeded in tissue culture-treated plates. The organoids were cultured with organoid growth medium (06030, STEMCELL) for 7-10 days before further experiments.

Isolation of liver hepatocytes

Primary hepatocytes were isolated using methods described previously[11,12]. The mouse livers were successively perfused with D-Hanks (CR-14175, Cienry) and type IV collagenase (17104-019, Gibco) solutions, and the hepatocytes were then dispersed by mechanical force. The suspensions were centrifuged at 30 × g for 3 minutes, and the precipitates obtained were hepatocytes.

Hepatic and cellular triglyceride assays

The contents of triglycerides (TGs) in liver tissue and hepatocytes were assayed using a commercial kit (E1013-105; Applygen). The liver tissues or collected cells were ground with lysis buffer. The appropriate amount of homogenate or lysate was then used for TG analysis, and the protein concentration in the remaining homogenate or lysate was quantified using the BCA Protein Assay Kit (P0010; Beyotime). The TG contents were normalized to equal protein concentrations.

Immunoprecipitation

HEK293T cells were transfected with FLAG-tagged full-length *HINT2*, HA-tagged full-length *SIRT3*, FLAG-tagged full-length *SIRT3*, and HA-tagged full-length *HINT2*. HA-tagged *SIRT3* (1-150 aa), HA-tagged *SIRT3* (151-300 aa), HA-tagged *SIRT3* (301-399 aa), HA-tagged *HINT2* (1-80 aa) or HA-tagged *HINT2*

(81-163 aa) was incubated for 48 hours and lysed with 1 ml of ice-cold IP lysis buffer (87788, Pierce, St Louis, MO, USA) and protease inhibitor cocktail (04693159001, Roche). Cell lysates were immunoprecipitated with 18 µl of anti-FLAG M2 affinity gel (A2220, Sigma) and incubated for 3 hours at 4°C with shaking (7.5 cycles/min). The agarose was then washed four times with lysis buffer and boiled in SDS sample buffer.

RNA-Binding Protein Immunoprecipitation

HepG2 cells and AML12 cells were seeded in a 15-cm dish and lysed with RIP lysis buffer (17-701, Millipore). Cell lysates were immunoprecipitated with YTHDF1 A/G magnet beads prepared with YTHDF1 antibody (ab220162, Abcam) and incubated for overnight at 4°C with shaking (7.5 cycles/min). The agarose was then washed with RIP wash buffer. The RNAs were purified, reversed transcription, and assessed by RT-PCR.

Protein extraction and immunoblotting

Proteins obtained using RIPA lysis buffer (FD008, FDbio) with protease inhibitor cocktail (04693159001, Roche) were quantified using the BCA Protein Assay Kit (P0010; Beyotime). Equal protein amounts were separated on SDS–polyacrylamide gels and transferred to PVDF membranes (Millipore). After blocking, the membranes were incubated overnight at 4°C with primary antibodies and then washed with secondary antibodies. The signal was visualized using an ECL light detection kit (Lianke Multi Sciences) and visualized using a ChemiScope 6000 Pro Touch (Clinx Science Instruments Co., Ltd., Shanghai, China). The antibodies used for Western blotting were the following: HINT2 (NOVUS, NBP1-86024 or LifeSpan, LS-C404041 for human; HUABIO, custom polyclonal antibody for mice), SIRT3 (CST; 5490), SLC25A51 (Abclonal; A21105), ACC (CST; 3662), p-ACC (Ser79) (CST; 3661), acetylated lysine (CST; 9441), OxPhos Rodent WB Antibody Cocktail (Invitrogen;45-8099), PDHA1 (Abcam; ab168379), SDHA (Abcam; ab137040), IDH2 (Abcam; ab131263), YTHDF1(abcam;ab220162), AMPKa (CST;2532), p-AMPKa(Thr172) (CST; 2531), Flag (Invitrogen; MA1-91878), HA (HUABIO; 0906-1), GAPDH (Proteintech; HRP-60004), and VDAC1 (HUABIO; ET1601-20).

RNA extraction and quantitative real-time PCR

RNA was isolated from tissue samples or cell lysates using the RNAiso (9108, Takara) reagent according to the manufacturer’s instructions and reverse transcribed

into cDNA using PrimeScript RT master mix (RR036, Takara). cDNA was extracted using SYBR Green (RR820, Takara) and a QuantStudio 5 system (Applied Biosystems) for quantitative real-time PCR. All target genes were normalized to the expression of GAPDH.

Immunofluorescence assay

Hepatocytes were fixed with 4% paraformaldehyde (Servicebio, Wuhan, China) and permeabilized with 0.5% Triton-X (Sangon, Shanghai, China). After blocking, the cells were incubated overnight at 4°C with primary antibodies (HINT2 (LifeSpan, LS-C404041 for human; HUABIO, custom polyclonal antibody for mice), SIRT3 (CST; 5490) , TOM20 (Santa Cruz; sc-17764)) and then with secondary antibodies (1 hour at RT), or at 37°C for 15 minutes with MitoTracker (M22425, Invitrogen), or at room temperature for 30 minutes with BODIPY 493/503 (GLPBIO, Montclair, CA) and phalloidin (ab176757, Abcam). Imaging was performed using the confocal microscope (FV3000, Olympus).

Mitochondrial membrane potential

Changes in the mitochondrial membrane potential (ΔΨm) were assessed using the JC-1-Mitochondrial Membrane Potential Assay kit (ab113850, Abcam) following the manufacturer’s protocol. The cells were incubated with 10 µM JC-1 dye in 1× dilution buffer for 10 minutes at 37°C in the dark. The JC-1 dye was then removed, then cells were washed once with 1× dilution buffer, and 100 µL of fresh 1× dilution buffer was added to each well. The red fluorescence (excitation/emission of 535 nm/590 nm) and green fluorescence (excitation/emission of 475 nm/530 nm) were measured using a SpectraMax i3x Microplate Reader (Molecular Devices). The ratio of red (aggregate) fluorescence to green (monomer) fluorescence was obtained.

NAD^+ detection

The NAD^+ concentration in hepatocytes was detected using an NAD/NADH kit (ab65348; Abcam). Following the manufacturer’s instructions, 2×10^6 hepatocytes were extracted with 400µl of $NADH/NAD^+$ extraction buffer by two freeze/thaw cycles and then centrifuged for 5 minutes at 4°C and top speed to remove the insoluble materials. The samples were added to a 10-kD Spin Column (ab93349, Abcam) and centrifuged at 10,000 × g and 4°C for 10 minutes. The filtrate was collected and mixed with NAD^+ Cycling Enzyme Mix for 5 minutes, and NAD^+ Developer was then added. Multiple readings were taken during 1~4 hours at OD 450

nm using a SpectraMax i3x Microplate Reader (Molecular Devices).

SIRT3 deacetylation activity

The deacetylation activity of SIRT3 was measured using a SIRT3 activity assay kit (ab156067, Abcam) following the manufacturer's protocol. Cells or tissues were lysed with lysate that did not contain protease inhibitors. Add ddH_2O, SIRT3 Assay Buffer, Fluoro-Substrate Peptide, NAD and Developer to 96 well microtiter plate (black wells) in order and mix well. Initiate reactions by adding the samples to each well and mixing thoroughly at room temperature. The fluorescent intensity was recorded for 60 minutes at 1 minute intervals using a SpectraMax i3x Microplate Reader (Molecular Devices) with excitation at 340-360 nm and emission at 440-460 nm and was normalized to the amount of total test protein.

Isolation of mitochondria and cytoplasm

Cytoplasmic and mitochondrial protein fractions were obtained using the Cell Mitochondrial Isolation Kit (C3601; Beyotime). Cells were collected and incubated with Mitochondrial Isolation Reagent for 10 minutes on ice and then homogenized 50 times with a glass homogenizer. After centrifugation at 600 × g and 4°C for 10 minutes, the supernatant was separated and centrifuged at 10,000 × g and 4°C for 15 minutes. The precipitate was the mitochondrial fraction, and the supernatant was the cytoplasmic fraction.

O_2 consumption rate (OCR)

For detection of the mitochondrial function in HepG2 cells and primary hepatocytes, we used the Agilent Seahorse XF Cell Mito Stress Test (103015-100, Agilent Technologies) according to the manufacturer's instructions. The kits for the assay included the regulators oligomycin (1.5μM), carbonyl cyanide 4-(trifluoromethoxy) phenylhydrazone (FCCP, 2.0μM) and rotenone/antimycin A (ROT/AA, 0.5μM).

Molecular analysis of publicly available datasets of MASLD patients

The data were obtained from the Gene Expression Omnibus (GEO) database (GSE48452 and 63067) (https://www.ncbi. nlm.nih.gov/geo/). A box plot was created using the R (version 4.1.1) software package ggplot2, and the correlation analysis was conducted using the package ggstatsplot.

Supplementary figures

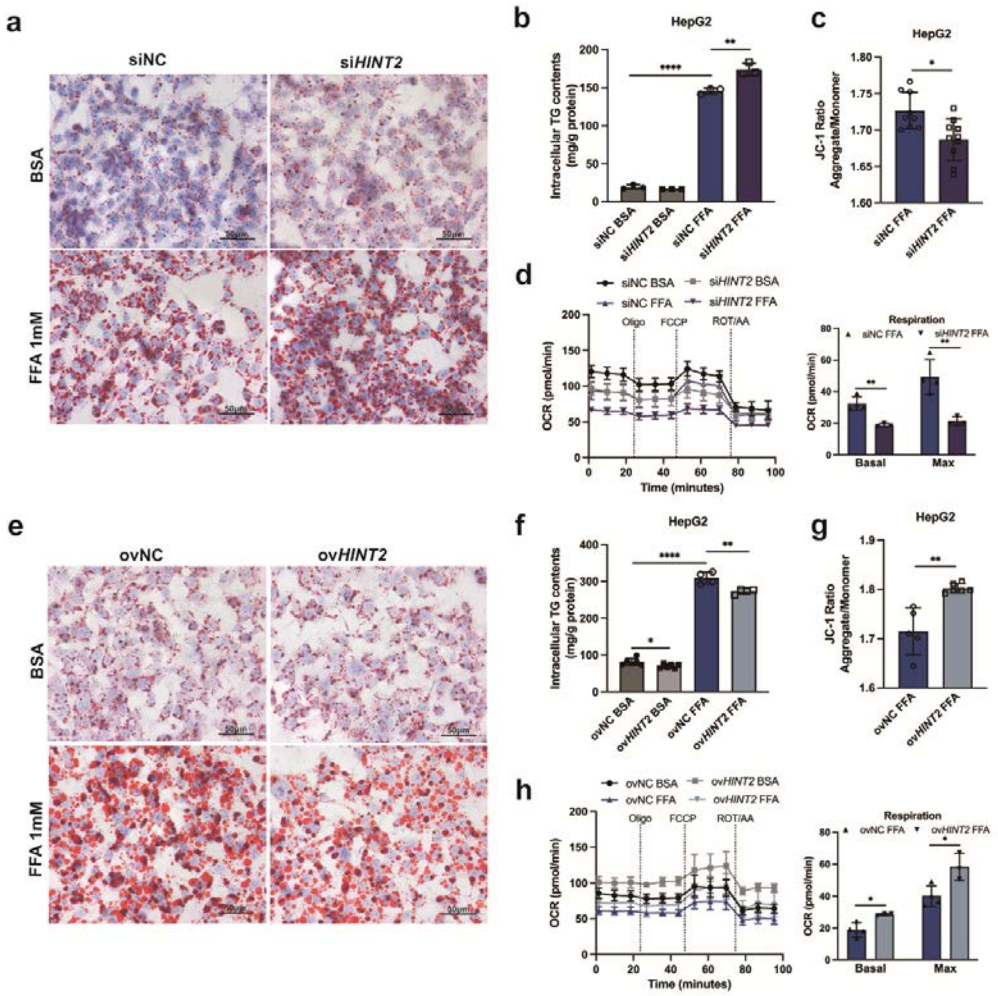

Supplementary Fig. 1. *Hint2* regulates mitochondrial function and lipid accumulation in FFA-stimulated HepG2 cells. HepG2 cells in which *HINT2* was silenced or overexpressed were treated with 1 mM FFA for 24 hours. (**a**) Oil red O staining. Scale bars: 50μm. (**b**) Intracellular TG contents. (**c**) JC-1 ratios. (**d**) OCR. (**e**) Oil red O staining. (**f**) Intracellular TG contents. (**g**) JC-1 ratios. (**h**) OCR. Data are presented as mean ± SD. $^{*}P < 0.05$, $^{**}P < 0.01$, $^{****}P < 0.0001$ (Student's *t*-test).

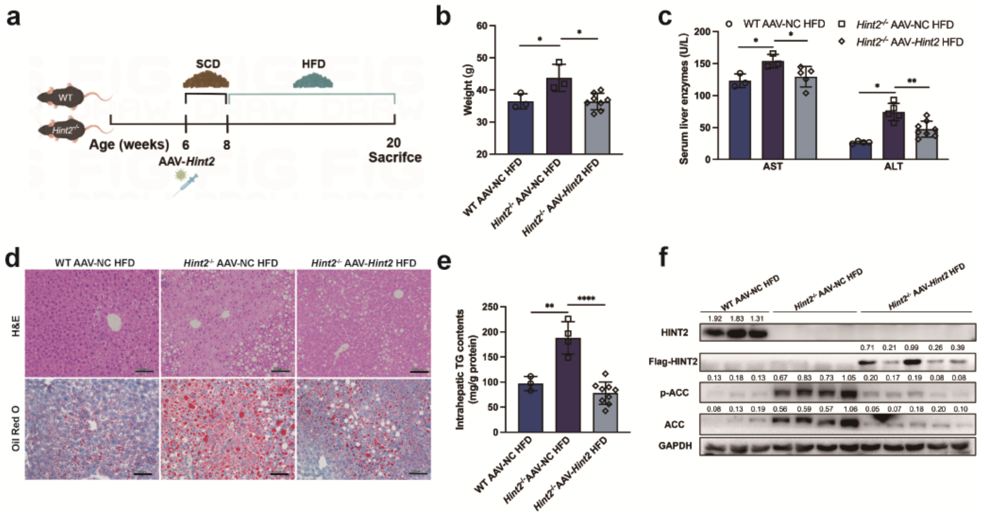

Supplementary Fig. 2. The overexpression of *Hint2* ameliorates HFD-induced MASLD in *Hint2*$^{-/-}$mice. (**a**) WT and *Hint2*$^{-/-}$ mice were injected with *Hint2*-overexpressing AAV through the tail vein and fed an HFD for 12 weeks. (**b**) Body weights of the mice. (**c**) Serum liver enzymes of the mice. (**d**) H&E and Oil red O staining of mouse liver sections. Scale bars: 100μm. (**e**) Intrahepatic TG contents of mice. (**f**) Western blot analyses of the protein levels of total and phosphorylated ACC in mouse livers. Data are presented as mean ± SD. $^{*}P < 0.05$, $^{**}P < 0.01$, $^{****}P < 0.0001$ (Student's *t*-test).

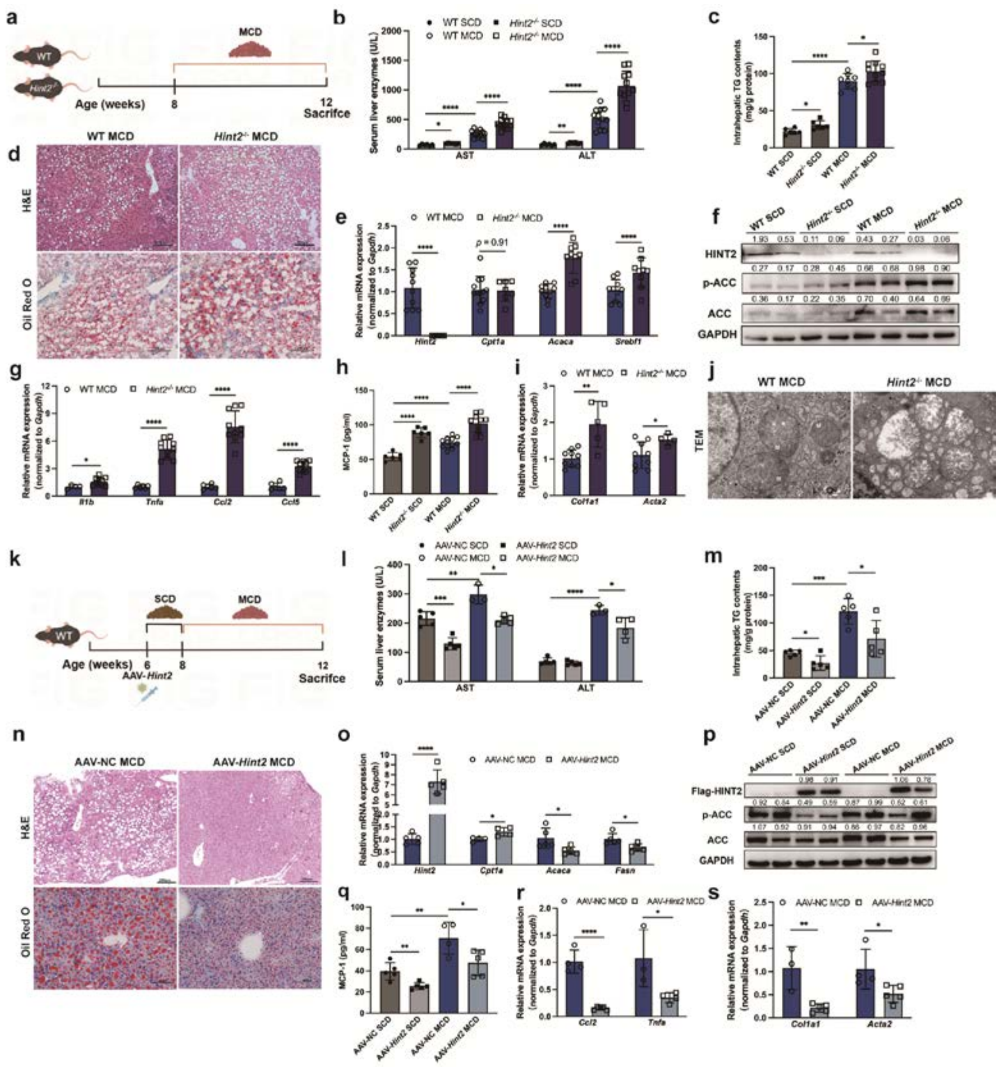

Supplementary Fig. 3. *Hint2* attenuates hepatic steatosis, inflammation, fibrosis, and mitochondrial damage in MCD-induced MASH mice. (**a**) WT and *Hint2*$^{-/-}$ mice were fed an MCD diet for 4 weeks. (**b**) Serum liver enzymes of the mice. (**c**) Intrahepatic TG contents of the mice. (**d**) H&E and Oil red O staining (scale bars: 100 μm) of mouse liver sections. (**e**) mRNA levels of *Cpt1a*, *Acaca* and *Srebf1*. (**f**) Protein levels of total and phosphorylated ACC. (**g**) mRNA levels of *Il1b*, *Tnfa*, *Ccl2* and *Ccl5*. (**h**) Serum MCP-1 concentrations of in mice. (**i**) mRNA levels of *Col1a1* and *Acta2* in mouse livers. (**j**) TEM (scale bars: 2μm) of mouse liver sections. (**k**) WT mice were injected with *Hint2*-overexpressing AAV through the tail vein and fed an MCD diet for 4 weeks. (**l**) Serum liver enzymes of the mice. (**m**) Intrahepatic TG contents of the mice. (**n**) H&E and Oil red O staining of mouse liver sections. Scale bars: 200μm. (**o**) mRNA levels of *Cpt1a*, *Acaca* and *Fasn*. (**p**) Protein levels of total and phosphorylated ACC. (**q**) mRNA levels of *Ccl2* and *Tnfa*. (**r**) Serum MCP-1 concentrations of in mice. (**s**) mRNA levels of *Col1a1* and *Acta2* in mouse livers. Data are presented as mean ± SD. $^{*}P < 0.05$, $^{**}P < 0.01$, $^{***}P < 0.001$, $^{****}P < 0.0001$ (Student's *t*-test).

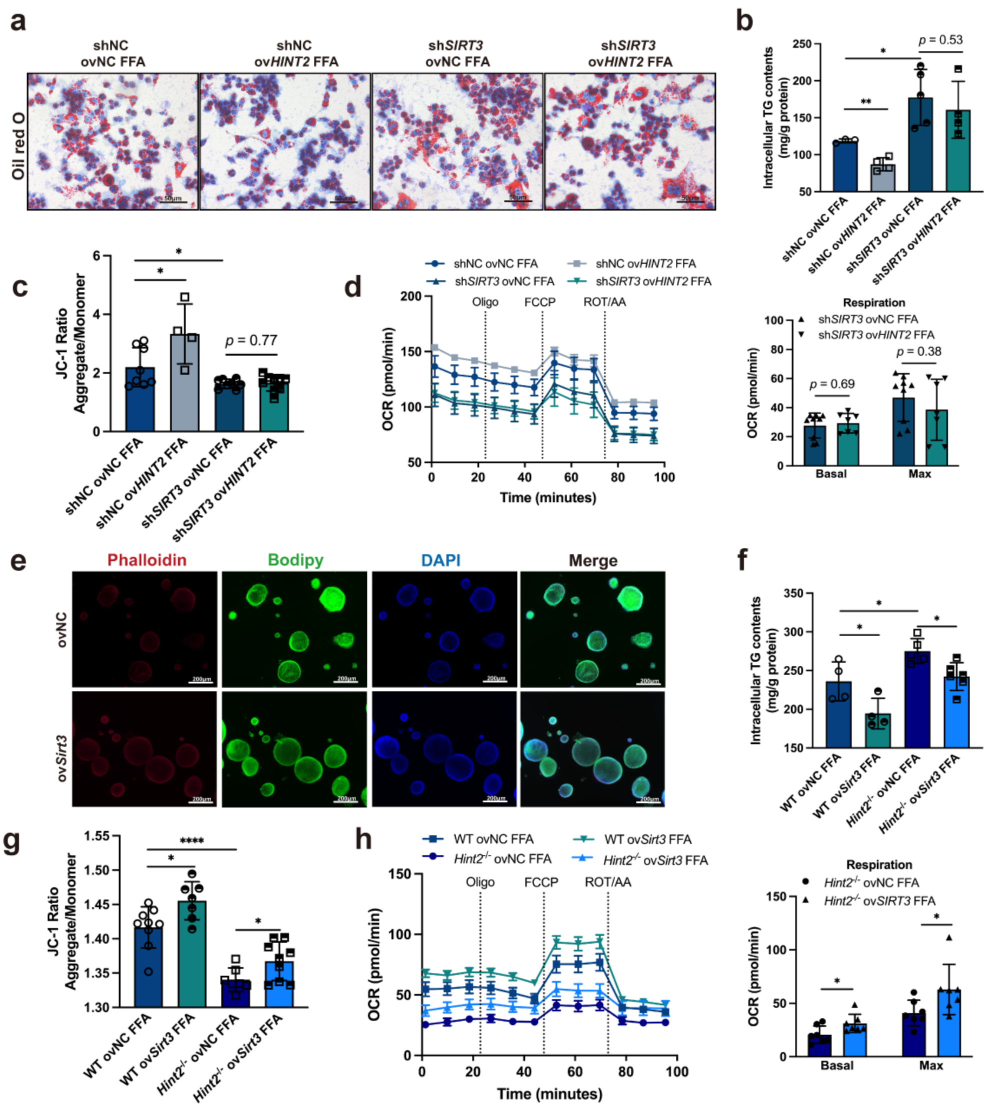

Supplementary Fig. 4. SIRT3 mediates the regulatory effects of HINT2 on FFA-induced lipid deposition and mitochondrial damage in hepatocytes. *HINT2*-silenced *SIRT3*-overexpressing HepG2 cells were further treated with 1 mM FFA for 24 hours. (**a**) Oil red O staining. Scale bars: 50μm. (**b**) Intracellular TG contents. (**c**) JC-1 ratios. (**d**) OCR. *Sirt3*-overexpressing $Hint2^{-/-}$ liver organoids or primary hepatocytes were treated with 200μM FFA for 48 hours or 1 mM FFA for 24 hours. (**e**) Lipid staining of $Hint2^{-/-}$ liver organoids with BODIPY. Scale bars: 200μm. (**f**) Intracellular TG contents. (**g**) JC-1 ratios. (**h**) OCR. Data are presented as mean ± SD. $^{*}P < 0.05$, $^{**}P < 0.01$, $^{****}P < 0.0001$ (Student's *t*-test).

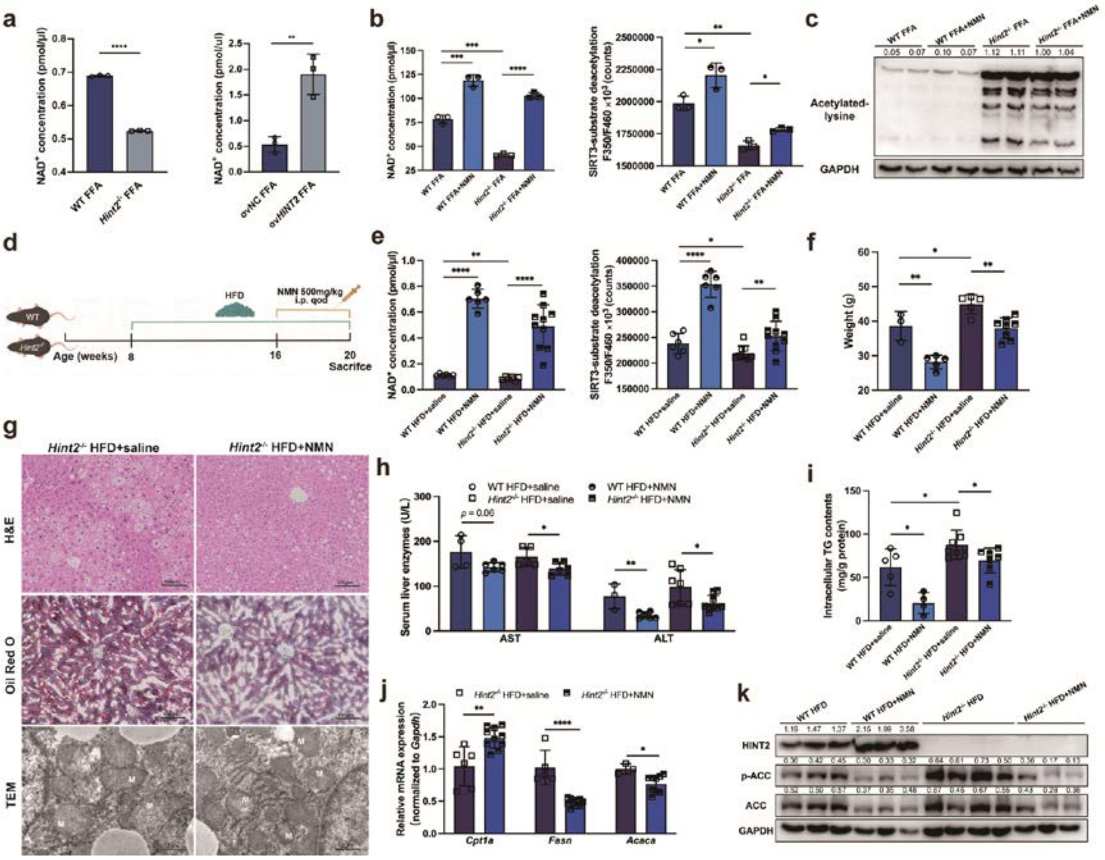

Supplementary Fig. 5. HINT2 regulates SIRT3 activity by modifying the NAD$^+$ concentration and thus affects MASLD. (**a**) NAD$^+$ concentrations in mitochondria of FFA-treated primary hepatocytes of *Hint2*$^{-/-}$ mice, and of FFA-treated ov*HINT2* HepG2 cells. (**b**) NAD$^+$ concentrations and SIRT3 activities in mitochondria of *Hint2*$^{-/-}$ primary hepatocytes treated with FFA and NMN. (**c**) Acetylation status of proteins in *Hint2*$^{-/-}$ primary hepatocytes treated with FFA and NMN. (**d**) WT and *Hint2*$^{-/-}$ mice were fed an HFD for 12 weeks and administered 500 mg/kg NMN by intraperitoneal injection for the last 4 weeks. (**e**) NAD$^+$ concentrations and SIRT3 activities in mitochondria of the mouse livers. (**f**) Body weights of the mice. (**g**) H&E, Oil red O staining (scale bars: 100μm) and TEM (scale bars: 2μm) of mouse liver sections. (**h**) Serum liver enzymes of the mice. (**i**) Intrahepatic TG contents of the mice. (**j**) qRT–PCR analyses of the mRNA levels of genes related to lipid synthesis (*Fasn* and *Acaca*) and *β*-oxidation (*Cpt1a*) in mouse livers. (**k**) Western blot analyses of the protein levels of total and phosphorylated ACC in mouse livers. Data are presented as mean ± SD. $^{*}P < 0.05$, $^{**}P < 0.01$, $^{***}P < 0.001$, $^{****}P < 0.0001$ (Student's *t*-test).

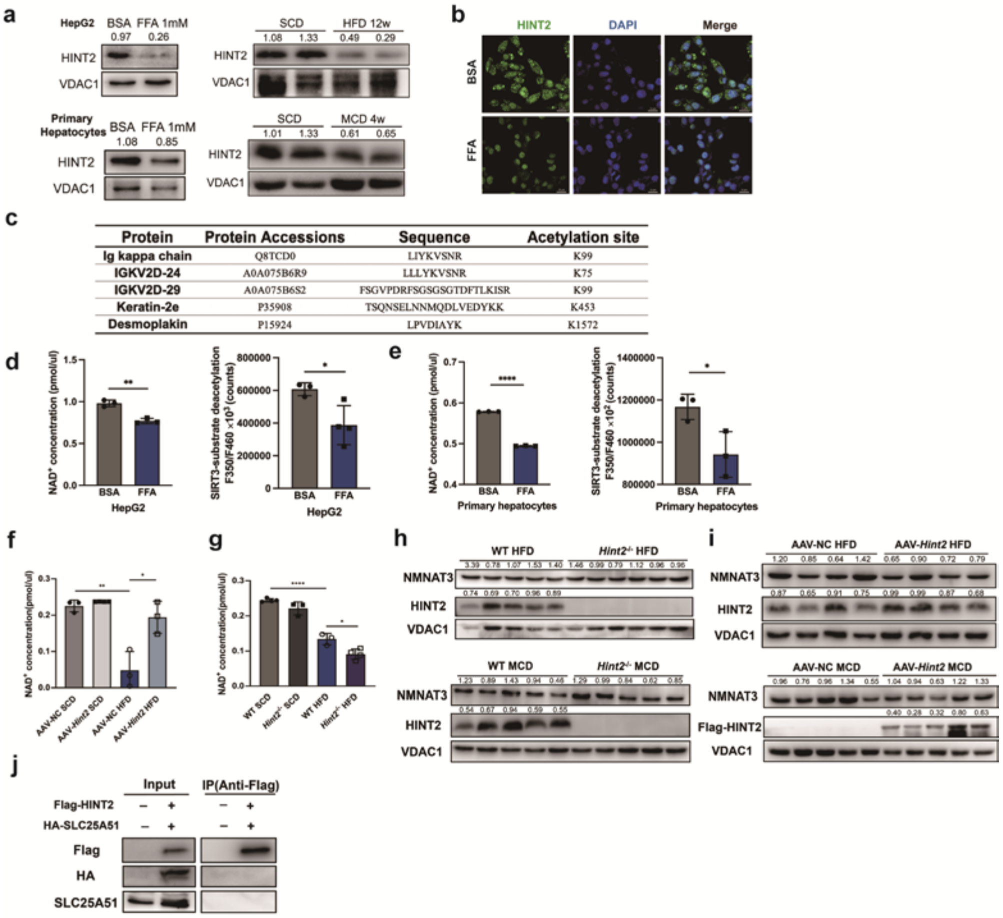

Protein	Protein Accessions	Sequence	Acetylation site
Ig kappa chain	Q8TCD0	LIYKVSNR	K99
IGKV2D-24	A0A075B6R9	LLLYKVSNR	K75
IGKV2D-29	A0A075B6S2	FSGVPDRFSGSGSGTDFTLKISR	K99
Keratin-2e	P35908	TSQNSELNNMQDLVEDYKK	K453
Desmoplakin	P15924	LPVDIAYK	K1572

Supplementary Fig. 6. Additional results. (**a**) Protein expression of HINT2 in the mitochondria of FFA-stimulated HepG2 cells or primary cells or in the mitochondria of HFD-fed mouse livers. (**b**) HINT2 expression in FFA-treated HepG2 cells. Scale bars: 10μm. (**c**) Mass spectrometric analysis of acetylated proteins pulled down by exogenous co-IP. (**d**) NAD^+ concentrations and SIRT3 activities in mitochondria of FFA-treated HepG2 cells. (**e**) NAD^+ concentrations and SIRT3 activities in mitochondria of FFA-treated primary hepatocytes. (**f and g**) Mitochondrial NAD^+ concentrations in the livers of HFD-fed AAV-*Hint2* and *Hint2*$^{-/-}$ mice. (**h**) Mitochondrial protein expression of NMNAT3 in the livers of HFD or MCD-fed wild-type and *Hint2*$^{-/-}$ mice. (**i**) Mitochondrial protein expression of NMNAT3 in the livers of HFD or MCD-fed control and *Hint2*-overexpressing mice. (**j**) Co-IP and Western blot analyses of the interaction between HINT2 and SLC25A51 in HEK293T cells. Data are presented as mean ± SD. $^{*}P < 0.05$, $^{**}P < 0.01$, $^{****}P < 0.0001$ (Student's *t*-test).
